# Analysis of Microsatellite Polymorphism in Inbred Knockout Mice

**DOI:** 10.1371/journal.pone.0034555

**Published:** 2012-04-11

**Authors:** Baofen Zuo, Xiaoyan Du, Jing Zhao, Huixin Yang, Chao Wang, Yanhua Wu, Jing Lu, Ying Wang, Zhenwen Chen

**Affiliations:** 1 Department of Laboratory Animal Science, School of Basic Medical Science, Capital Medical University, Beijing, China; 2 Model Animal Research Center, Nanjing University, Nanjing, China; Emory University School of Medicine, United States of America

## Abstract

Previously, we found that the genotype of 42 out of 198 mouse microsatellite loci, which are distributed among all chromosomes except the Y chromosome, changed from monomorphism to polymorphism (CMP) in a genetically modified inbred mouse strain. In this study, we further examined whether CMP also relates to the homologous recombination in gene knockout (KO) mouse strains. The same 42 microsatellite loci were analyzed by polymerase chain reaction (PCR) in 29 KO inbred mouse strains via short tandem sequence repeat (STR) scanning and direct sequence cloning to justify microsatellite polymorphisms. The C57BL/6J and 129 mouse strains, from which these 29 KO mice were derived, were chosen as the background controls. The results indicated that 10 out of 42 (23.8%) loci showed CMP in some of these mouse strains. Except for the trinucleotide repeat locus of D3Mit22, which had microsatellite CMP in strain number 9, the core sequences of the remaining 41 loci were dinucleotide repeats, and 9 out of 41 (21.95%) showed CMPs among detected mouse strains. However, 11 out of 29 (37.9%) KO mice strains were recognized as having CMPs. The popular dinucleotide motifs in CMP were (TG)_n_ (50%, 2/4), followed by (GT)_n_ (27.27%, 3/11) and (CA)_n_ (23.08%, 3/13). The microsatellite CMP in (CT)_n_ and (AG)_n_ repeats were 20% (1/5). According to cloning sequencing results, 6 KO mouse strains showed insertions of nucleotides whereas 1 showed a deletion. Furthermore, 2 loci (D13Mit3 and D14Mit102) revealed CMP in 2 strains, and mouse strain number 9 showed CMPs in two loci (D3Mit22 and D13Mit3) simultaneously. Collectively, these results indicated that microsatellite polymorphisms were present in the examined inbred KO mice.

## Introduction

Microsatellites, or short tandem sequence repeats (STRs), are highly polymorphic repetitive DNA sequences 1–6 base pairs (bp) in length [1] that are randomly distributed throughout eukaryotic genomes [2]. Due to their abundance within a genome, random occurrence, and high degree of polymorphisms [3], STRs are ideal tools for deciphering genetic variability. Basic repeat units of microsatellites vary from one to a few thousand base pairs [4], [5]. Microsatellites are useful for detecting genomic DNA damage and/or mutational events, e.g., deletions, insertions, and point mutations [6], based on the polymorphisms that they contain. This alteration, demonstrated by the insertion or deletion of repeat units, was first observed in colorectal tumors [7]–[11]. According to the nature of the repetitive DNA sequences within them, microsatellites are particularly prone to slippage during DNA replication, leading to variations in their size defined as microsatellite instability (MSI).

MSI, being the result of a deficiency in DNA mismatch repair (MMR) systems [12], [13], which include the hMLH1, hMSH2, hMSH3, hMSH6, hPMS1and hPMS2 genes [14]–[23], is caused by germ-line and/or somatic mutations. Thus, MSI can be used as a molecular marker to detect defective DNA MMR [14], [15], [24], [25]. For example, 5 microsatellite markers, including the mononucleotide markers BAT 25 and BAT 26 as well as the dinucleotide markers D2S123, D5S346, and D17S250, have been recommended by the National Cancer Institute workshop for MSI detection in colorectal cancer [25], [26]. Thirteen STR loci are stored in the Combined DNA Index System, one of the most recent developments in forensic DNA technology, because of their “high discrimination potential”, which can identify 99% parent-child kinships [27]. In addition, it is useful for monitoring inbred-mouse genetic quality because over 99% of the microsatellite loci in inbred mice theoretically show monomorphisms.

Gene knockout (KO) is an effective tool for analyzing gene function and generating model animals. Since the first KO mouse was reported in 1989 [28], KO mice have been popular in biomedical sciences and have yielded more than 50,000 mutants, as can be found in PubMed [29], [30]. However, the functional relevance of gene targeting has been questioned because the mutation via gene targeting may lead to an avalanche of compensatory processes (up- or down-regulating the gene products), resulting in secondary phenotypic changes. Nevertheless, a mutant phenotype is often less severe than expected and/or detectable only in a subset of the tissues [29]. One explanation for this is the possible existence of functional redundancy between genes and compensatory mechanisms between gene family members [31]. Unfortunately, few reports have elucidated the relationship between MSI- and KO-induced secondary phenotypic changes thus far. Previously, we investigated 198 mouse microsatellite markers that are distributed among all chromosomes, except the Y chromosome, in genetically modified (transgenic and spontaneously mutated) inbred mice (unpublished data). Interestingly, compared with the background strain controls, 42 loci were found to have changed from monomorphism to polymorphism (CMP). To determine the relationship between MSI- and KO-phenotypic changes, we employed 42 identified MSI-sensitive loci to investigate whether CMP could be detected in KO mouse strains. The results supported our hypothesis.

## Materials and Methods

### Sample collection

The freshly frozen liver tissues of 29 KO mouse strains derived from congenic inbred C57BL/6J (B6) strains were obtained from either the Model Animal Research Center, Nanjing University, or the Institute of Laboratory Animal Science, Chinese Academy of Medical Sciences. The mice were maintained in a normal 12 h/12 h light/dark cycle with regular mouse chow and water ad libitum at an AAALAC-accredited specific pathogen-free (SPF) facility. IVC (individually ventilated cages) were used for breeding all mice within a barrier facility (Figure S2). Animal welfare and experimental procedures were performed under the supervision of the Institutional Animal Care and Use Committee (IACUC), strictly in accordance with the guidelines for the care and use of laboratory animals (NRC, 1996 and 2011; FASS 2010 and ETS123). Embryo cryopreservation was performed after the mice's arrival, and we renewed the core colony every three years. Genetic quality control was performed using SNP and microsatellite maker screening every year.

The sources and IDs of the 29 KO mouse strains are shown in Table 1. The donor embryonic stem (ES) cells of these KO strains were derived from the 129 strains. The B6 mice that were obtained from the Jackson Laboratory (Bar Harbor, ME) in 2008 served as the hosts. The chimeric mice were usually mated to B6 mice for 10 generations to obtain homozygous KO mice. To minimize the background genetic influence, the liver tissues from both non-modified B6 and 129 mice were used as the background controls for each KO mouse strain. In addition, the liver tissues from 10 non-modified B6 mice belonging to 3 consecutive generations were used to detect the genetic stability of the 42 loci in the background controls.

**Table 1:** The information about 29 knock-out mouse strains.

No	ID	Name	Origin*
1	D0104	IRG-47.KO	N
2	J004781	B6·129P2-Lyz2<tm1(cre)>Ifo/J	N
3	J003288	B6·129S7-Ifngr1^tm1Agt^/J	N
4	J002055	B6·129P2-Apoa1^tm1Unc^/J	N
5	J002753	B6·129S6/SvEvTac-Atm^tm1Awb^/J	B
6	J002118	B6·129P2-Tcrb^tm1Mom^/J	N
7	J002052	B6·129P2-Apoe^tm/Unc^/J	N
8	J002216	B6.129S7-Rag1^tmiMom^/J	N
9	D0045	LRG-47KO	N
10	J002287	B6·129S7-Ifng^tm1Ts^/J	N
11	J006096	B6·Cg-Msr1<tm1Csk>/J	N
12	J002207	B6·129S7-Ldlr^tm1Her^_/J_	N
13	J004650	B6·129-Tlr2^tmIkir/J^	N
14	J005670	B6·CgGt(ROSA)26Sor<tm1(rtTA,EGFP)Nagy>/J	N
15	J002684	B6·129P2-Nos3^tm1Unc^/J	N
16	J0106440	B6·129S4-Pten^t**m1**Hwu^/J	N
17	J002251	B6·129P2-Il10^tm1Cgn^/J	N
18	J003288	B6·129S7-Ifngr1^tm1Agt^/J	B
19	J002207	B6·129S7-Ldlr^tm1Her^/J	B
20	J002287	B6·129S7-Ifng^tm1Ts^/J	B
21	J002609	B6·129P2-Nos2^tm1Lau^/J	B
22	J002684	B6·129P2-Nos3^tm1Unc^/J	B
23	J007251	B6·129-Mapt^tm1Hnd^/J	B
24	J006367	129-Cckar^tm1Kpn^/J	B
25	J002849	B6·129P-Nfkb1^tm1Bal^/J	B
26	J002770	B6·129S2-Cd40lg^tm1Imx^/J	B
27	J006148	B6·129X1-Gt(ROSA)26Sor^tm1(EYFP)Cos^/J	B
28	J002216	B6·129S7-Rag1^tm1Mom^/J	B
29	J004781	B6·129P2-Lyz2^tm1(cre)Ifo^/J	B

* N: Model Animal Research Center of Nanjing University, B: Institute of Laboratory Animal Science, Chinese Academy of Medical Sciences.

### DNA Extraction

The genomic DNAs were extracted from the frozen liver tissue samples of all KO mice and their corresponding controls by using a standard phenol-chloroform extraction and ethanol precipitation method, as previously described [31]. The amount of DNA from each sample was determined by measuring the A260/A280 value using a microplate absorbance reader system (Bio-Rad 680, USA) and further evaluated by agarose gel electrophoresis. The DNA was diluted to a concentration of 100 ng/µL and stored at −20°C for later use as the template for PCR.

### Microsatellite Analysis

First, the genetic stability of the 3 consecutive generations B6 mice was examined using 10 mice. In brief, the DNAs of 10 B6 non-modified mice, including 3 (No. 1-3) parental-generation, 3 (No. 4-6) first-generation and 4 (No. 7-10) second-generation mice, were extracted. Forty-two microsatellite loci were examined using PCR for each mouse, and the results were evaluated using STR scanning and DNA sequencing. The primer sequences and the optimized annealing temperatures for each locus are shown in Table S1. To efficiently perform STR scanning, the forward primer of each locus was tagged at the 5′ ends with 3 types of fluorescent markers, FAM, HEX, or TAMRA. Then, 42 loci with CMP in the genetically modified mice were used to evaluate the microsatellite polymorphisms in this study.

For PCR, the reaction samples were each prepared to a total volume of 20 µL as follows: 2 µL 10× buffer, 0.5 µmol/L each primer, 125 µmol/L dNTPs, 1.0 U Taq DNA polymerase, 1.5–2.5 mmol/L MgCl_2_, and 100 ng template DNA. The PCR was performed in a gradient thermal cycler (BIO-RAD Inc. ALS1296) in accordance with the following protocol: pre-denaturation at 94°C for 5 min; 35 cycles of denaturation at 94°C for 30 s, annealing at an optimized temperature as shown in Table S1 for each microsatellite for 30 s, and extension at 72°C for 30 s; and a final extension at 72°C for 7 min was performed. The amplification of the PCR products was confirmed on 2% agarose gels stained with ethidium bromide and was visualized using a UV transilluminator (Vil Ber Lou RMAT Inc.).

Three kinds of PCR products that were synthesized from fluorescently labeled primers were mixed at a ratio of 1∶2∶3 (FAM: HEX: TAMRA). A total of 1 µL of each mixture was gently mixed with 25 µL formamide, which was then visualized using capillary electrophoresis (CE) on an ABI-3730XL DNA Analyzer system (PE Biosystems, USA). The peak height of the waves for each product was determined using GeneMarker software. For sequencing, the PCR products (total volume of 50 µL) were first purified using an ABI BigDye Terminator v3.1 Cycle Sequencing Kit, cloned into the PMD18-T Vector (TAKARA), and then sequenced using an ABI 3730XL DNA Sequencer.

## Results

### Detection of genetic variations in 3 consecutive generations of B6 mice

Both STR scanning data (Figure S1) obtained in our study supported the concept that none of the sequences of the 42 microsatellite loci had changed in all 3 consecutive generations of B6 mice. In addition, the genotypes of all these loci were homologous and consistent.

### Microsatellite polymorphism analysis by STR scanning

**Figure 1:**
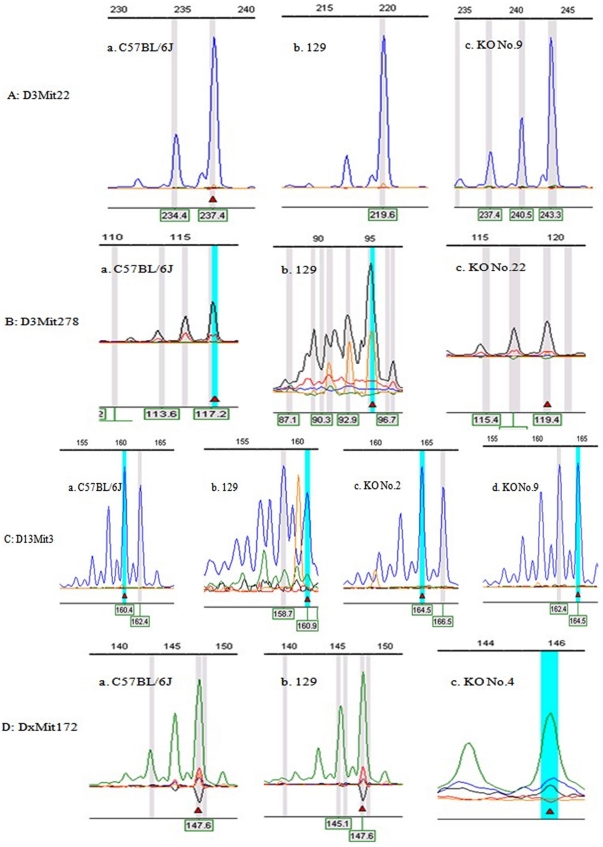
STR loci D3Mit22 (A), D3Mit278 (B), D13Mit3 (C), and DxMit172 (C) showed microsatellite polymorphisms in knockout mice number 9, 22, 2 and 9, 4 compared to wide-type C57BL/6J and 129 mice, respectively. The allele size of wide-type C57BL/6J, 129, and knockout mice number 9 at locus D3Mit22 are 237 bp (A-a), 220 bp (A-b), and 243 bp (A-c), respectively. The allele size of wide-type C57BL/6J, 129, and knockout mice number 22 at locus D3Mit278 are 117 bp (B-a), 95 bp (B-b), and 119 bp (B-c), respectively. The allele size of wide-type C57BL/6J, 129, and knockout mice number 2 and 9 at locus D13Mit3 are 162 bp (C-a), 161 bp (C-b), 165 bp (C-c) and 165 bp (C-d), respectively. The allele size of wide-type C57BL/6J, 129, and knockout mice number 4 at locus DxMit172 are 148 bp (D-a), 148 bp (D-b), and 146 bp (D-c), respectively.

After scanning the PCR products of 42 loci, several CMPs were detected in parts of the genomes of the mouse strains at different loci. An overview of the STR scanning results is shown in Table 2, and the typical figures demonstrating the CMPs at given loci such as D3Mit22, D3Mit278, D13Mit3 and DxMit172 are shown in Figure 1. In brief, 10 STR loci (10/42, 23.8%) were found to have CMPs (Table 2) in these mouse strains, among which the CMPs of 8 loci (D3Mit22 (KO No. 9), D3Mit51 (KO No. 5), D3Mit278 (KO No. 22), D10Mit266 (KO No. 23), D8Mit14 (KO No. 8), D11Mit 258 (KO No. 16), D12Nds2 (KO No. 17) and DxMit172 (KO No. 4)) existed in only one strain. Notably, 2 STR loci had CMP in 2 mouse strains simultaneously, such as D13Mit3 in the KO mouse strains numbered 2 and 9 and D14Mit102 in the KO mouse strains numbered 28 and 29. In addition, we found 11 (37.9%) of the 29 KO mouse strains showed microsatellite CMP in this study. In KO mouse strain number 9, two loci (D3Mit22 and D13Mit3) had polymorphic changes.

**Table 2:** The microsatellite markers showing polymorphisms in knockout mice, including the name of microsatellite markers and knockout mice strains, background control, and repeat motif changes.

STR marker	Allele size				No. of KO, AN of CS	STR of KO mice (compared to control)		CS of KO mice (compared to control)	
	C57BL/6J		129			C57BL/6J	129	C57BL/6J	129
	STR	CS, AN	STR	CS, AN					
D3Mit22	237	237, JN410560	220	219, JN410559	9, JN410561	243(+6)	243(+23)	+2 (GCT)	+8(GCT)
D3Mit51	239	239, JN410563	246	246, JN410562	5, JN410564	264(+25)	264(+18)	+12 (GT)	+9(GT)
D3Mit278	117	110, JN410566	95	88, JN410565	22, JN410567	119(+2)	119(+24)	+1(TG)	+12(TG)
D8Mit14	139	143, JN410569	164	165, JN410568	8, JN410570	141(+2)	141(−23)	+1(CG)	−10(CA)
D10Mit266	93	92, JN410573	87	88, JN410572	23, JN410574	98(+5)	98(+11)	−3(CA)	−1(CA)
D11Mit258	129	127, JN410579	155	153, JN410578	16, JN410580	168(+39)	168(+13)	+14(GT)+1(AT)	+2(GT)
D12Nds2	193	189, JN410583	172	169, JN410582	17, JN410584	197(+4)	197(+25)	+1(TG)	+10(TG)+1(TA)
D13Mit3	162	161, JN410586	161	161, JN410585	2, JN410587	165(+3)	165(+4)	+2 (GT)	+2(GT)
					9, JN410588	165(+3)	165(+4)	+1(CT)	+1(CT)
D14Mit102	120	119, JN410590	139	137, JN410589	28, JN410592	124(+4)	124(−15)	+1(CA)	−6(CA)−1(CT)−1(TA)
					29, JN410591	124(+4)	124(−15)	+2(CA)	−5(CA) +1(TA)
DxMit172	148	146, JN410597	148	146, JN410596	4, JN410598	146(−2)	146(−2)	−2(TG)	−2(AG)

KO means knockout mice; STR means the results of short tandem repeat scanning; CS means cloning sequences; “+” (insertion), “−” (deletion); the number behind “+” or “−” indicates nucleotides and the letter enclosed in the parenthesis indicates core sequence repeats. AN means the accession number on GenBank.

### Microsatellite polymorphism analysis by cloning sequence

To further confirm the above results of the STR scanning, the PCR products of all microsatellite loci having CMPs were cloned into a plasmid (PMD18-T Vector, TAKARA) and sequenced via a BigDye Terminator v3.1 Cycle Sequencing Kit (ABI) on the 3730XL automatic DNA sequencing system. To ensure that stuttering of TAQ polymerase effects have been excluded in this study, we compared the DNA sequence supplied by these commercial companies to the existing reports on the NCBI website. The blast results between ours and the reports on the website matched each other well. For examples, after blasting our sequence result of locus D3Mit22 (237 bp) with the sequence upload on the NCBI website, either the size, flank sequence or the core sequences were perfectly matched to each other (Table S1).

All of the sequenced results and the accession numbers of either wild type mice (B6 and 129) or KO mice are also shown in Table 2. The results indicated that the CMPs detected via STR scanning matched consistently with the sequenced data. Therefore, each sequence of the 11 loci with CMPs in the KO mouse strains differed from those of the background controls.

Among all 42 STR loci, the core sequence of D3Mit22 was characterized by a trinucleotide repeat and showed CMP in KO mouse strain number 9. The core sequences of the remaining 41 loci were dinucleotide repeats that contained the core sequence of either an (AC)_n_, (TG)_n_, (GT)_n_, (AG)_n_, (CA)_n_ or (CT)_n_ motif. In addition, all of the loci with dinucleotide motifs totalled a CMP ratio of 21.95% (9/41). The most popular dinucleotide motif with CMP was (TG)_n_ (50%, 2/4) followed by (GT)_n_ (27.27%, 3/11) and (CA)_n_ (23.08%, 3/13). The rest of the dinucleotide repeats (AG)_n_ and (CT)_n_ showed a CMP ratio of 20% (1/5) and 20% (1/5), respectively. Notably, D3Mit22, D3Mit51, D3Mit278, D11Mit258, D12Nds2 and D13Mit3 in 6 KO mouse strains (numbered 9, 5, 22, 16, 17 and 2, respectively) were characterized by an existing insertion of core nucleotides, and locus DxMit172 was characterized by an existing nucleotide deletion.

**Figure 2:**
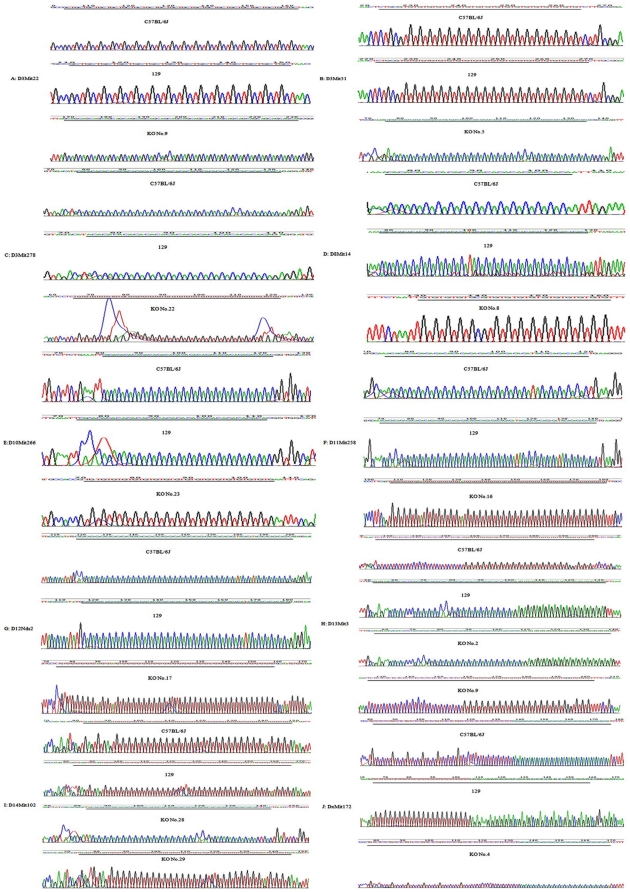
Cloning sequences show microsatellite polymorphism in 10 loci (from up to low is the sequence for C57BL/6J, 129, and the knockout mouse (KO), respectively). From figure A to J shows the sequences of locus D3Mit22, D3Mit51, D3Mit278, D8Mit14, D10Mit266, D11Mit258, D12Nds2, D13Mit3, D14Mit102, DxMit172 in turns. The underlined section indicates the repeat region. In each figure through A–J, the red lines indicate thymine, black lines indicate guanine, blue lines indicate cytosine and green lines indicate adenine.

The sequences of 10 CMP loci in the corresponding B6, 129, and KO mouse strains are shown in Figure 2. Interestingly, locus D8Mit14 in KO mouse strain number 8 showed an insertion (+1(CG)) of core nucleotides as compared to the B6 control strain, but these strains showed a nucleotide deletion (−10(CA)) as compared to the 129 control strain. Locus D14Mit102, with a compound class of repeats, showed an insertion (+1(CA)) compared to B6 but a deletion (−6(CA)−1(CT)1(CT)) compared to the 129 strain numbered 28; however, an insertion (+2(CA)) compared to B6 as well as a deletion (−5(CA)) and an insertion (+1(TA)) compared to the 129 strain numbered 29 was also found.

## Discussion

In this study, we investigated the frequency of microsatellite CMP in 29 KO mice using 42 STR markers. The results indicated that there existed microsatellite CMP differences between KO mice and their corresponding controls.

To exclude the influence of genetic variance from sampling, we examined the microsatellite stability at 42 loci in 10 non-modified B6 mice that belonged to 3 consecutive generations. As expected, there were no variations among all 3 generations of mice at every microsatellite loci, and the genotype of these loci was homologous. The results implied that the B6 mice used in this study as the background controls of the KO mice were reliable in their genetic aspect.

As reported, a genetic background (the ES cell or donor) with an induced mutation affects the phenotype of the homozygous mutant mouse (KO mouse) [31]–[33]. The KO mouse strains used in our study were produced via applying 129-derived ES cells as the mutant donor. The mosaic mice were backcrossed at least 10 generations to a corresponding B6 strain to generate KO/congenic strains. The KO inbred mouse can be considered genetically identical to the recipient (B6), except for the following genomic elements: the donor locus of interest (intentionally introgressed), some genes linked to that locus (unintentionally introgressed), and random genetic elements from the donor genome (also unintentionally introgressed). In other words, the genomic changes in the B6 strain related only to the donor and the KO gene locus. In this circumstance, to justify the derivation of the existing CMP in the KO mice, the two contributors of the resulting KO mice, non-modified B6 and 129 mice, were chosen as the background controls to simultaneously conduct every single STR scan and DNA sequencing.

In our study, the most frequently occurring microsatellite CMP existed in the loci where the dinucleotide repeat was (TG)_n_, displaying at a frequency of 75%, (3/4); the second was (AG)_n_ at 40% (2/5). However in humans and rats, the most abundant microsatellite was (AC)_n_ [3]. Interestingly, Y. Xue et al. studied CMP in tuberculosis (TB) patients and suggested that the S/M genotype [≤GT16 (S allele); GT17 to GT22 (M allele)] of the microsatellite (GT)_n_ polymorphisms in intron 2 of the Toll-like receptor 2 gene may increase the susceptibility to TB in Chinese people [34]. Therefore, it is possible that the locus containing the core sequence of GT repeats in our study may be sensitive to gene knockout. However, this hypothesis requires further validation.

Importantly, we found that D3Mit51 showed CMP in KO mouse strain number 5 (Atm KO Chr9:53247254-53344845 bp). The gene Atm encodes ATM, which is a member of the PI-3 kinase protein family, and it plays a crucial role in the signal transduction network that modulates cell-cycle checkpoints, DNA repair, and other cellular responses to DNA damage. This gene is also responsible for ataxia telangiectasia (AT) [35]. The Atm KO mice present an osteopenic phenotype as early as 10 weeks old, apparently due to decreased bone marrow-derived mesenchymal progenitors. As previously stated, the femur length shows significant linkage with loci on proximal chromosome 3, which contains D3Mit51 [36]. Therefore, we speculate that the KO Atm gene may influence the stability of D3Mit51, which is on the same chromosome as the Atm gene.

Our research also indicated that the other two loci, D3Mit22 (Chr 3:69522098-69522334) and D3Mit278 (Chr3:71816030-71816141 bp), on chromosome 3 also showed CMPs. D3Mit22 is linked to both the ribosomal protein L32 pseudogene, the function of which is thus far unknown, and Tshp4 (tooth shape 4), which may be involved in the osteopenic phenotype (http://www.ncbi.nlm.nih.gov/genome/sts/sts.cgi?uid=116286). In addition, D3Mit278 linked mainly to a DNA segment that did not show any linkage with the KO genes LRG-47 and Nos3 (http://www.ncbi.nlm.nih.gov/genome/sts/sts.cgi?uid=129706).

B. Ledermann noted that genes closely linked to the disrupted locus could influence the phenotype of the induced mutation, which in turn complicates the interpretation of the results [29]. To support this hypothesis, we analyzed the linkage relationship between microsatellites and KO genes; however, no linkage was found among them. In addition, we analyzed the relationship between the microsatellite loci with CMPs and their linked genes; however, no functional relationship seems to exist between the microsatellite loci-linked genes and the knockout genes. The reasons remain elusive.

Collectively, these results indicate that microsatellite polymorphisms exist in the examined inbred KO mice. However, we are still unsure as to what type of relationship exists between MSI and KO mice.

## Supporting Information

Figure S1 **STR scanning results of 10 B6 control mice belonged to 3 consecutive generations.**
(DOC)

Figure S2 **Mice used in this study were maintained on a normal 12 h/12h light/dark cycle with regular mouse chow and water ad libitum at an AAALAC accredited specific pathogen-free (SPF) facility.** IVC cages were used for all mice within barrier facility.
(DOC)

Table S1 **Comparation of sequence of locus D3Mit22 between published data and ours.** Green colors indicate primer sequences, red colors indicate core sequence of the locus.
(DOC)
