# Supplementary material for: Analysis of Microsatellite Polymorphism in Inbred Knockout Mice
Source: PLoS One. 2012 Apr 11;7(4):e34555. doi: 10.1371/journal.pone.0034555 (PMC3324499; doi:10.1371/journal.pone.0034555)
Supplement: Figure S1 — STR scanning results of 10 B6 control mice belonged to 3 consecutive generations. (DOC) [file pone.0034555.s001.doc]

**Supplemental materials**

Figure 1. STR scanning results of 42 loci in 10 B6 mice (background control strain in this study) from 3 consecutive generations. At each locus, the size of the locus was shown. Number 1-3 indicated the scanning results of parental mice, 4-6 indicated the first generation and 7-10 indicated the second generation.

**
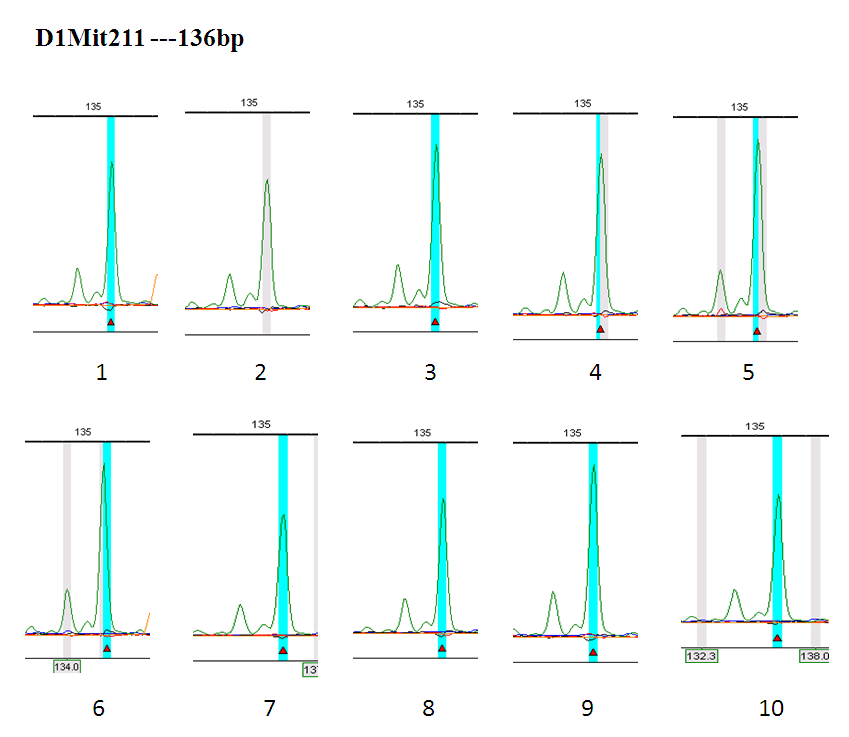
**

**
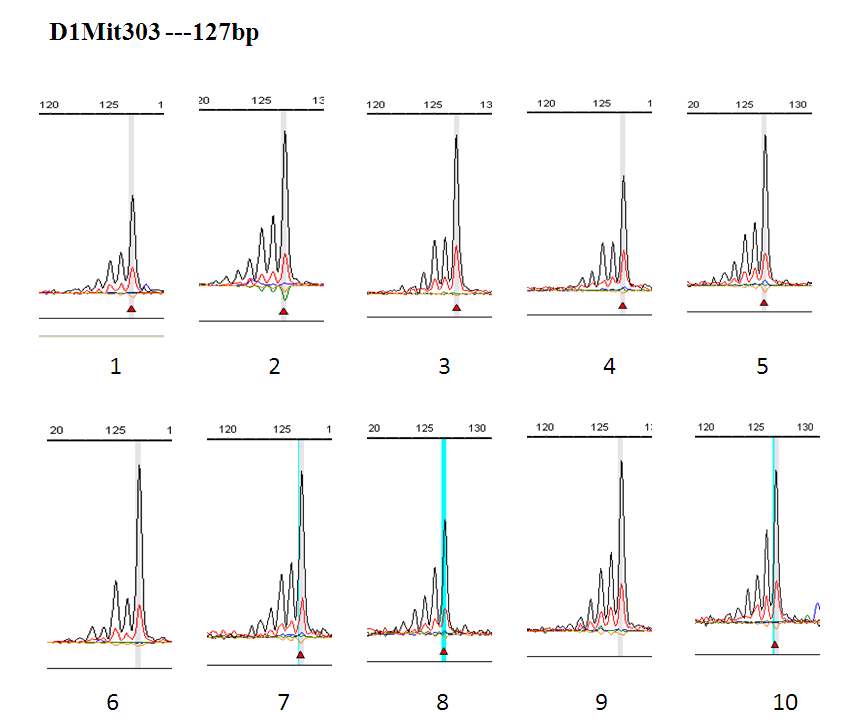
**

**
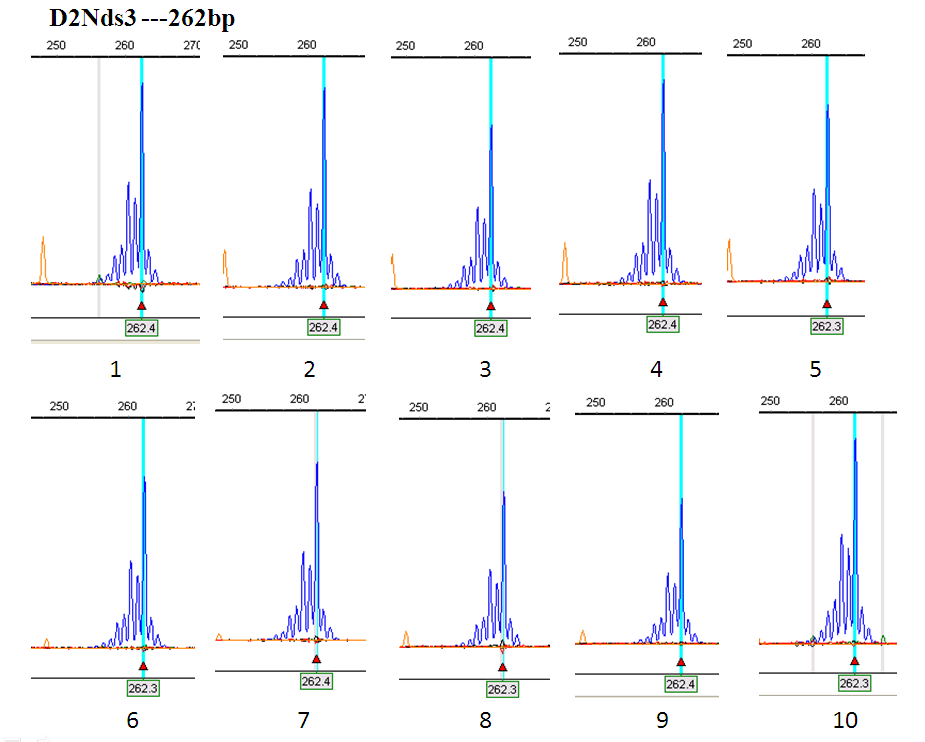
**

**
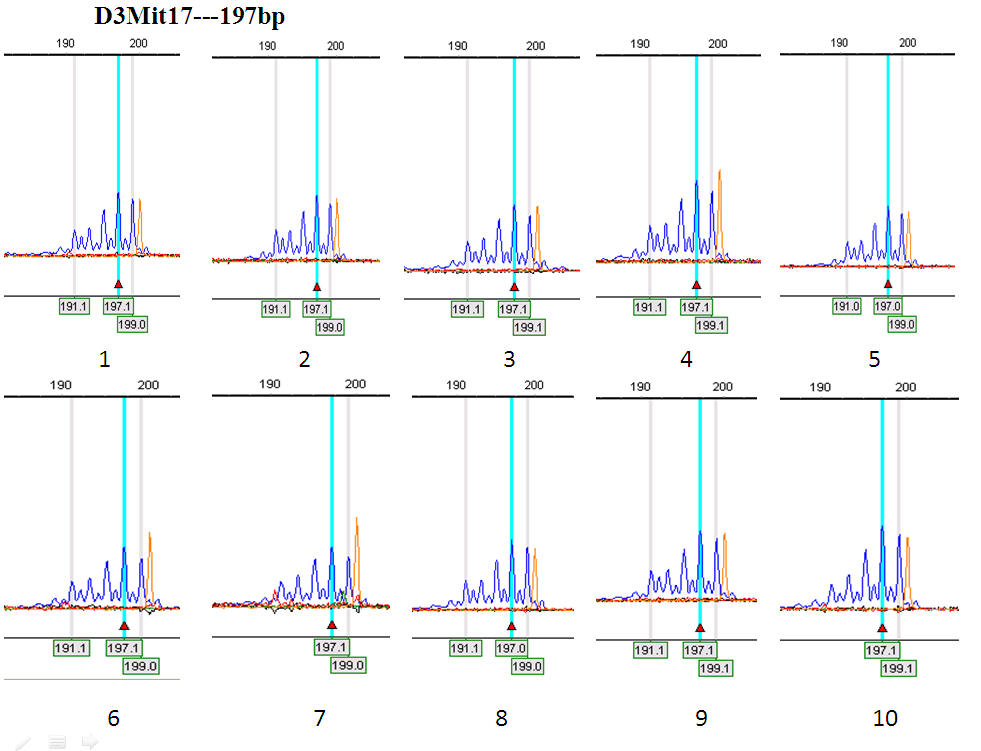

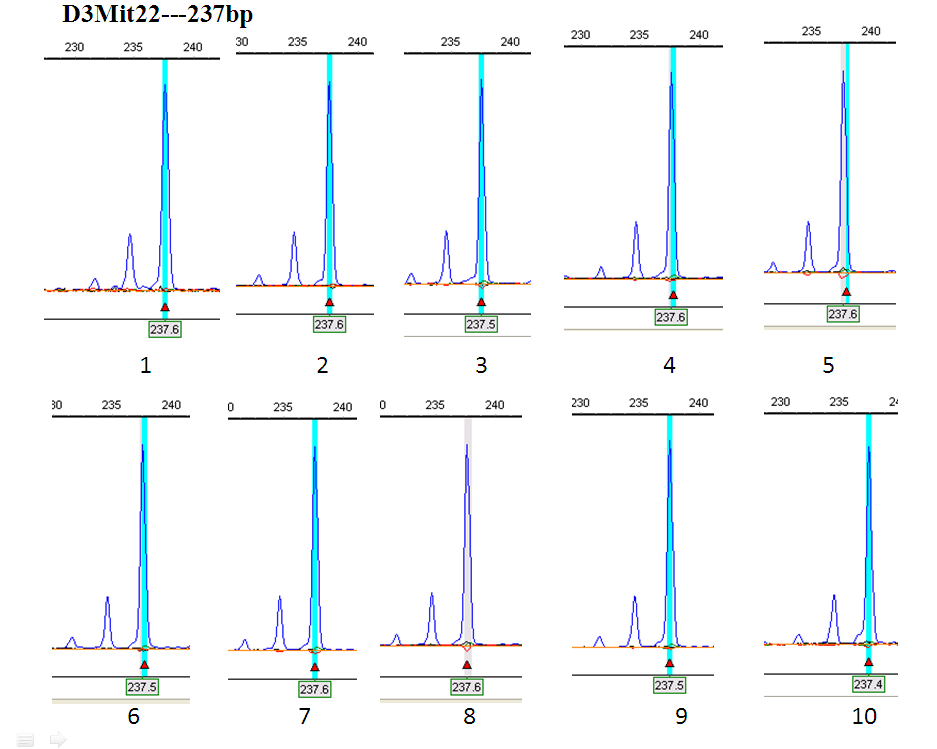

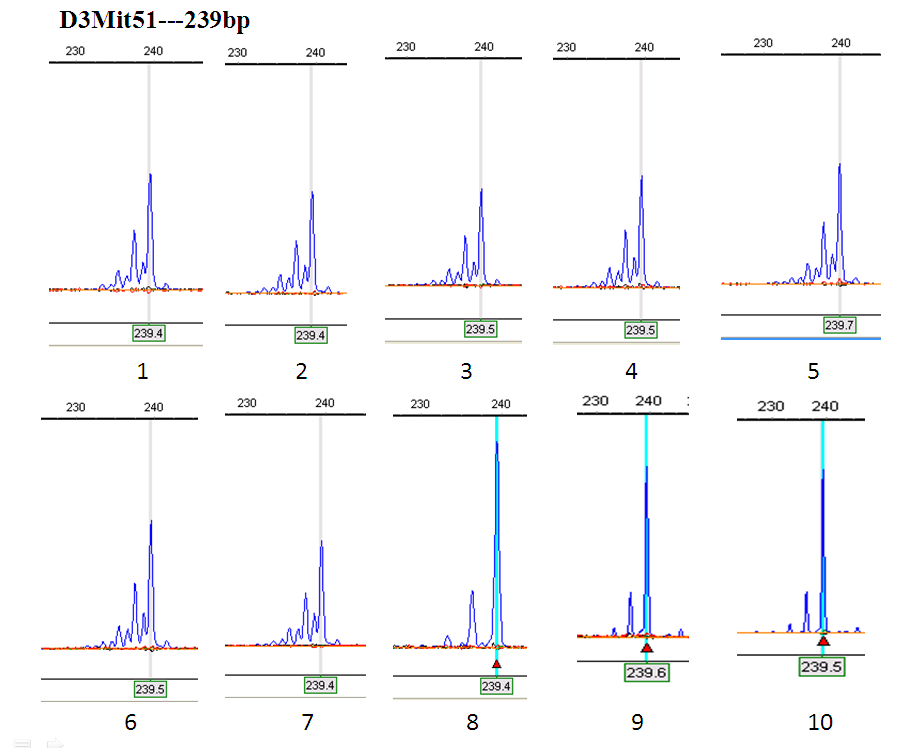

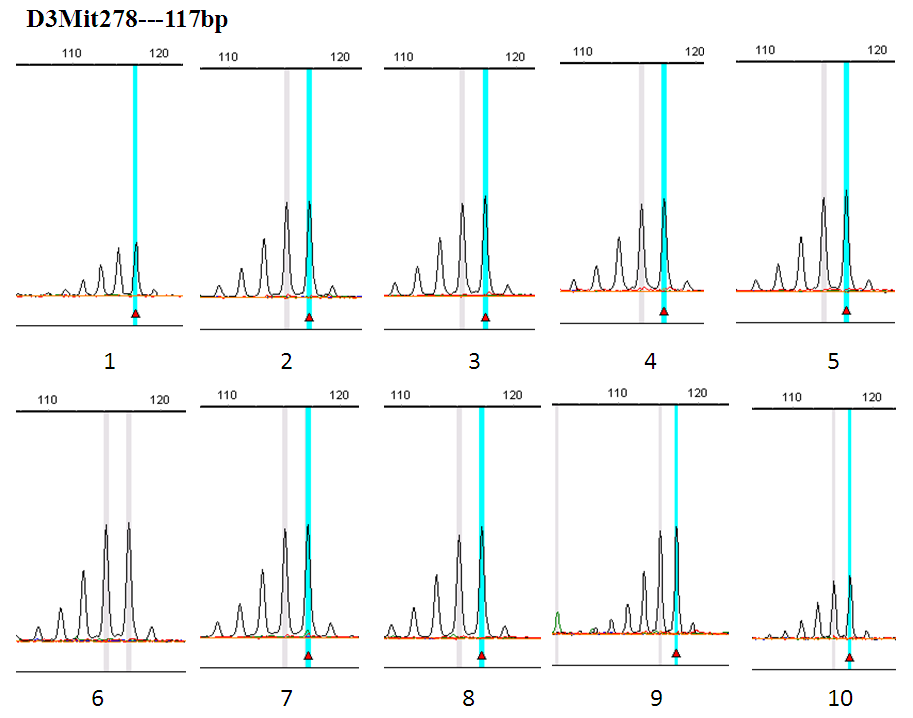

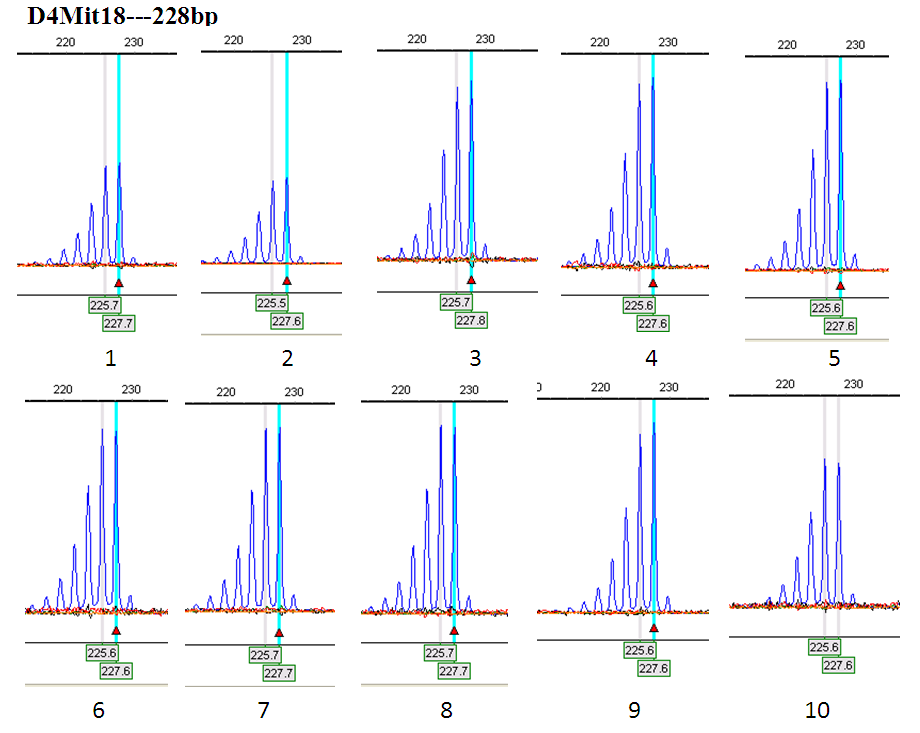

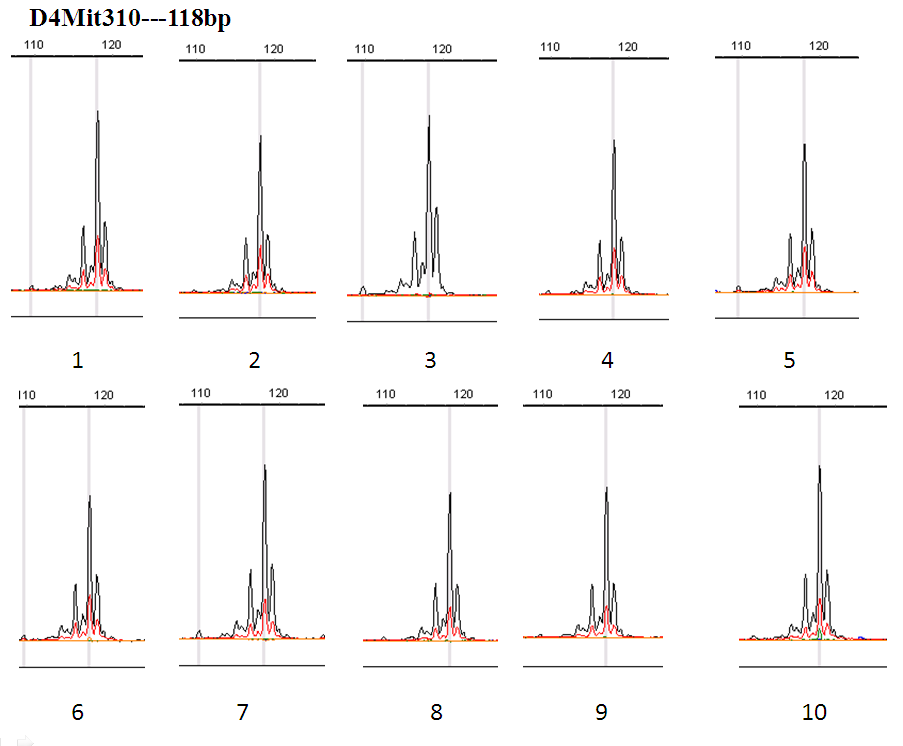

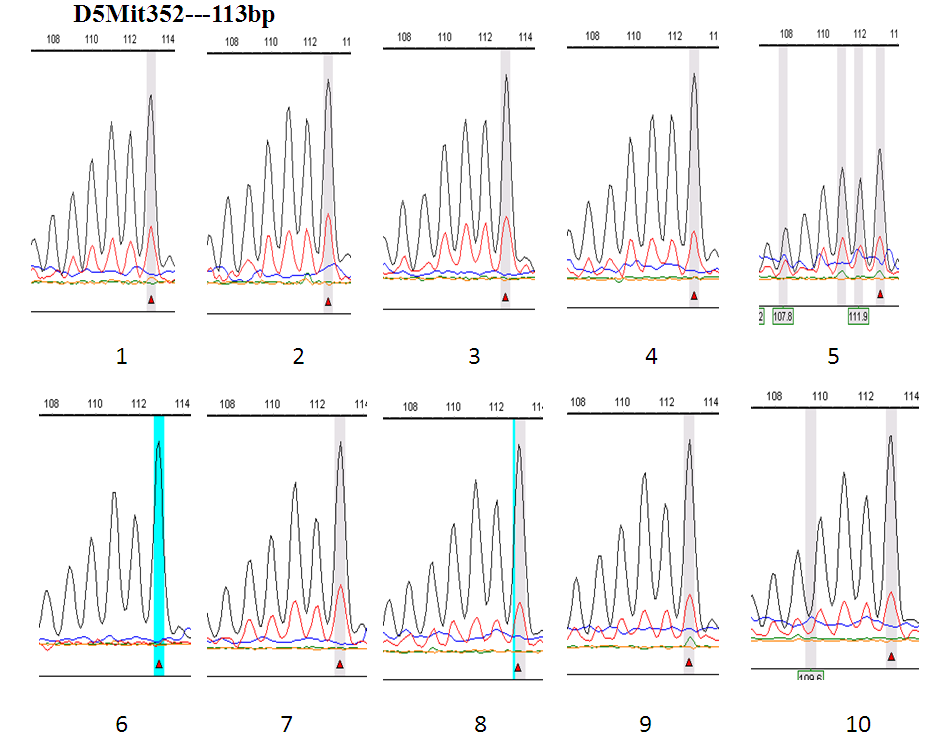

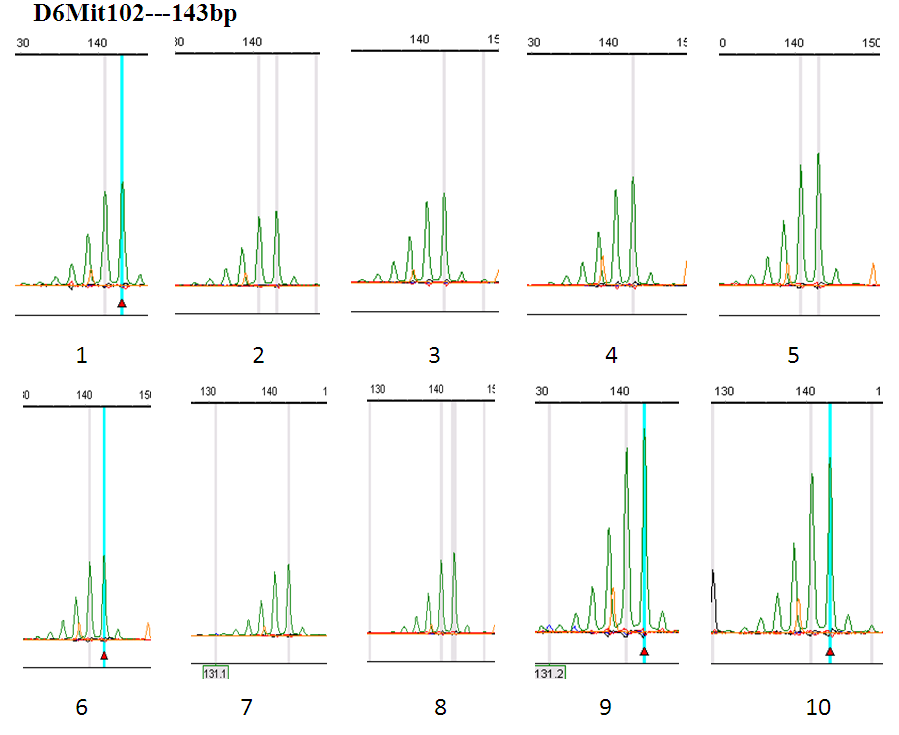

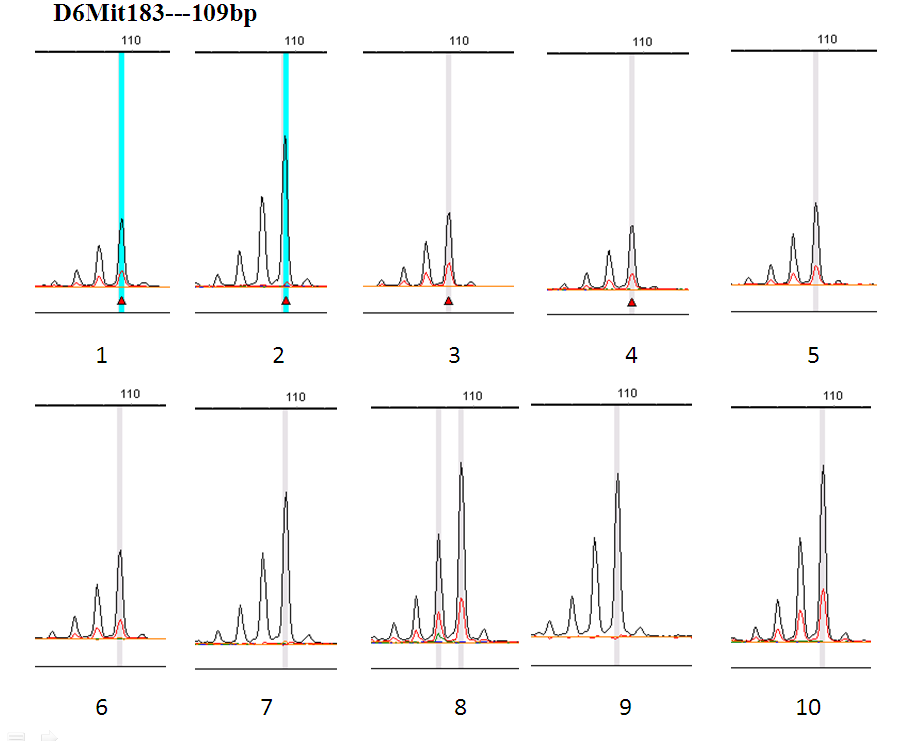

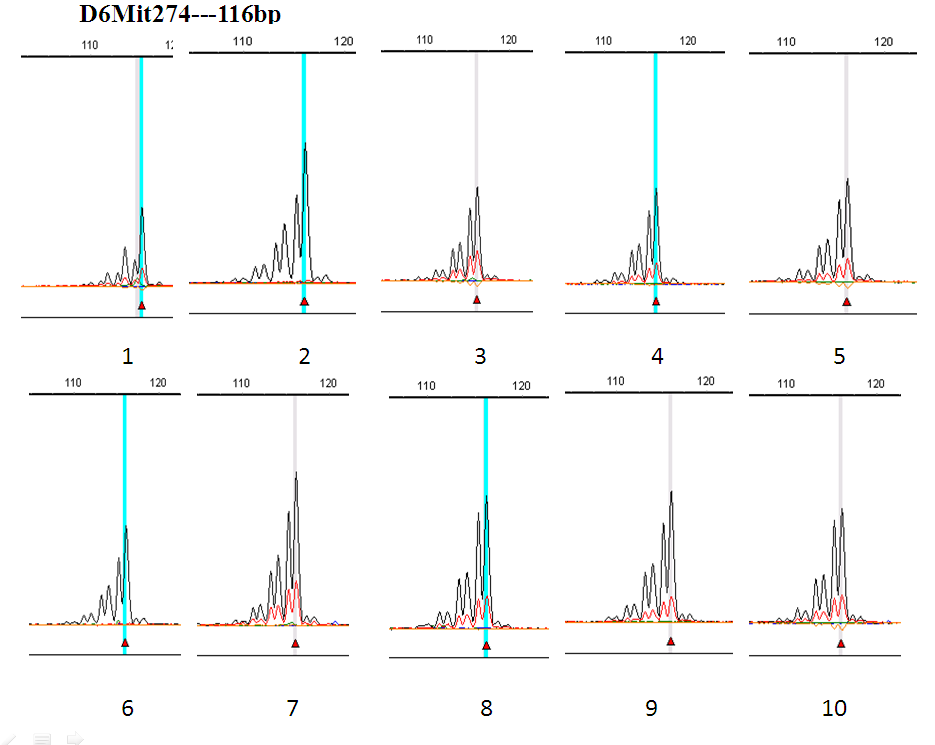

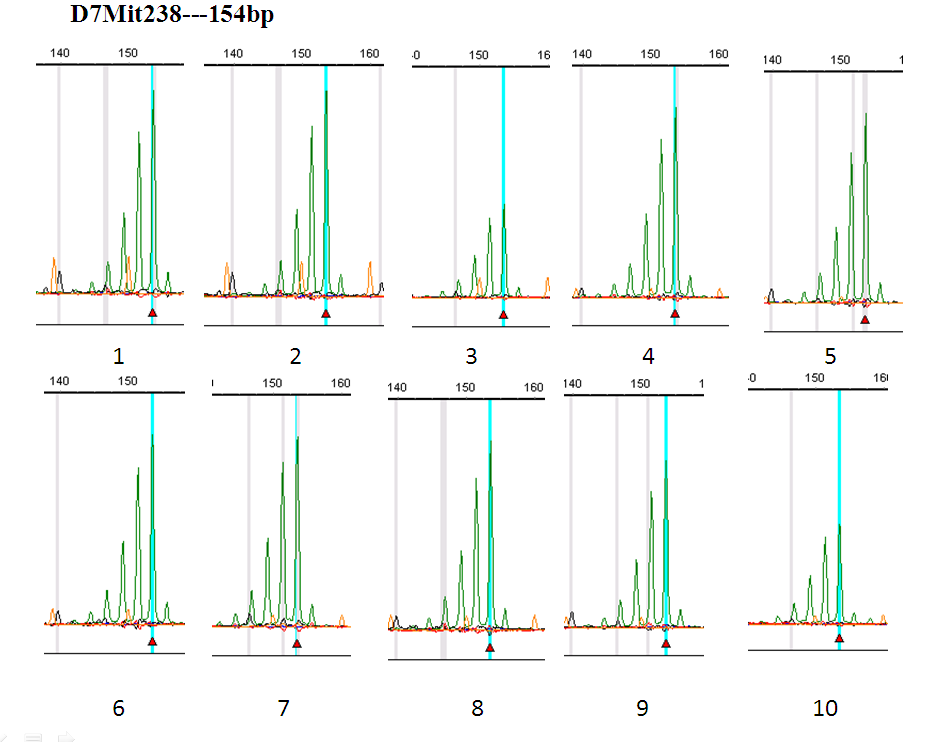

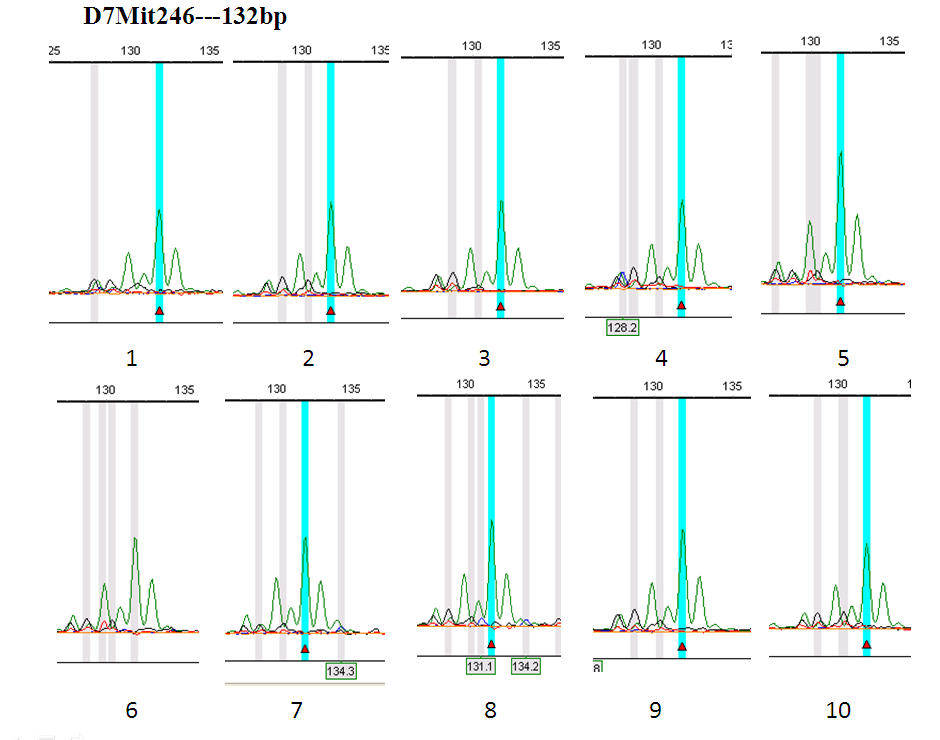

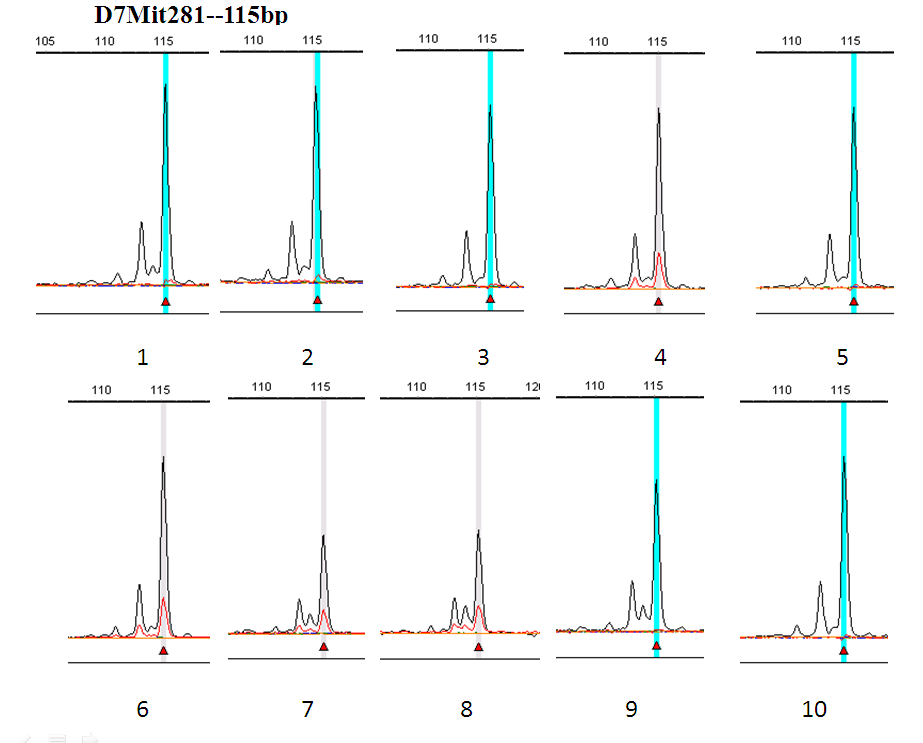

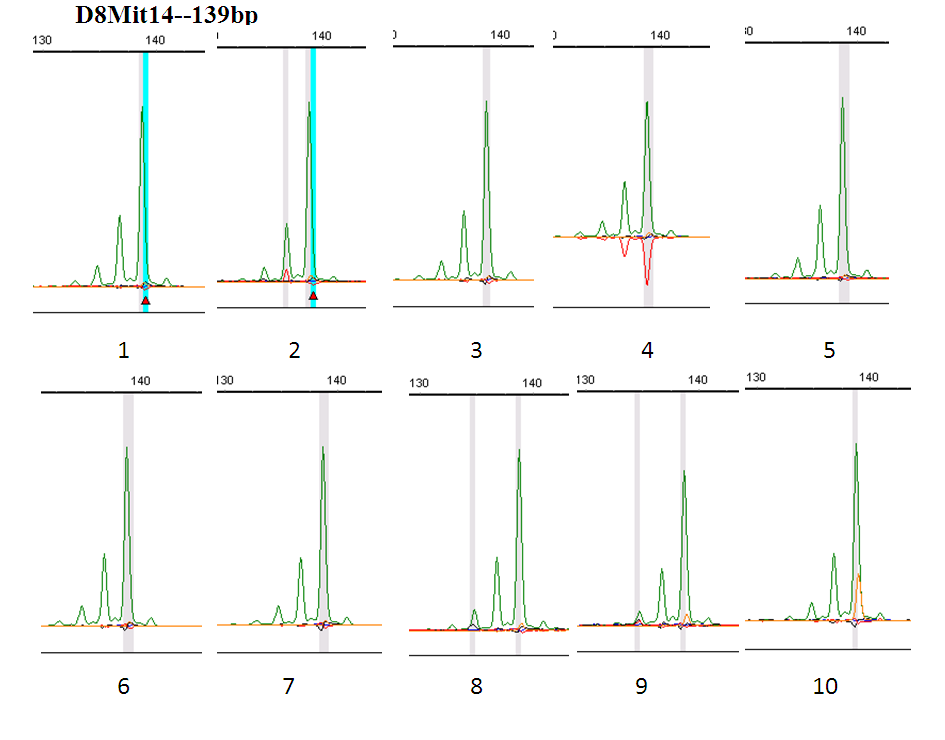

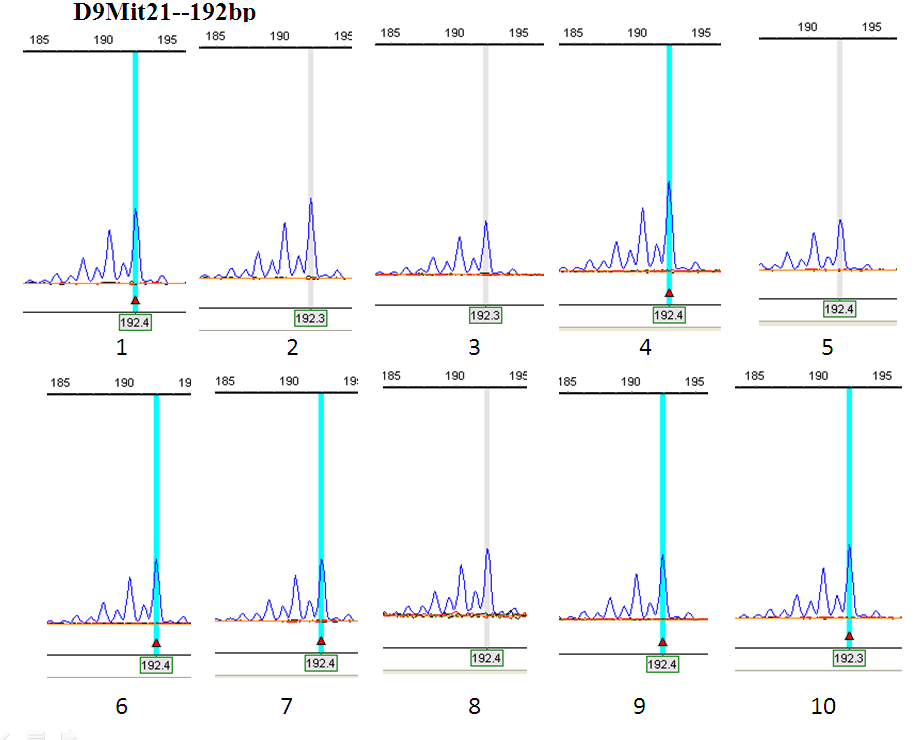

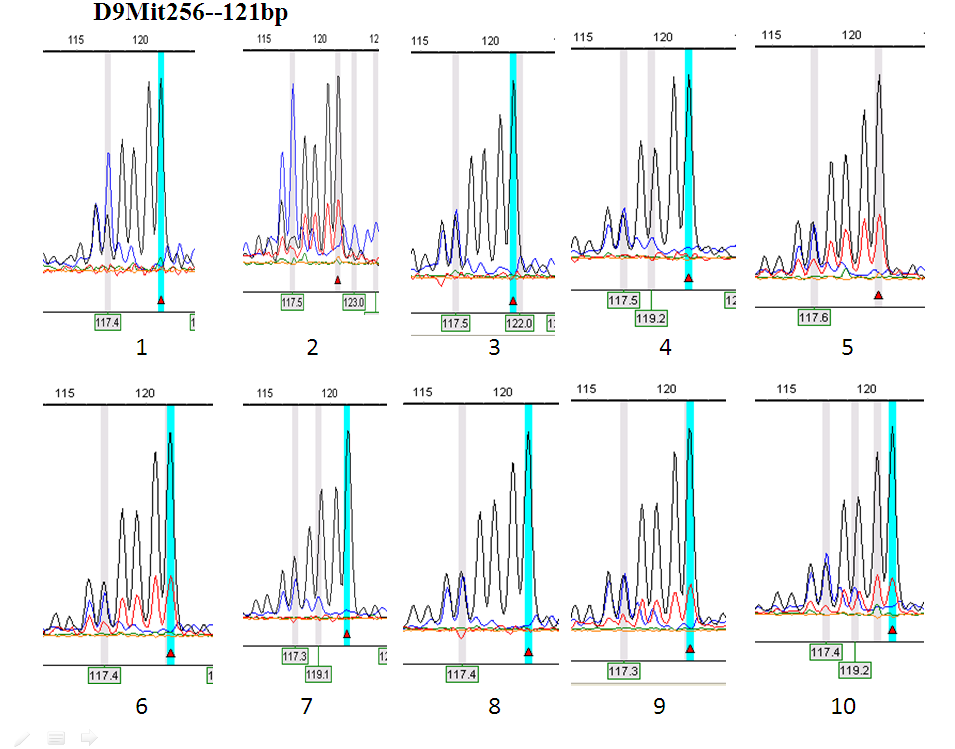
**

**
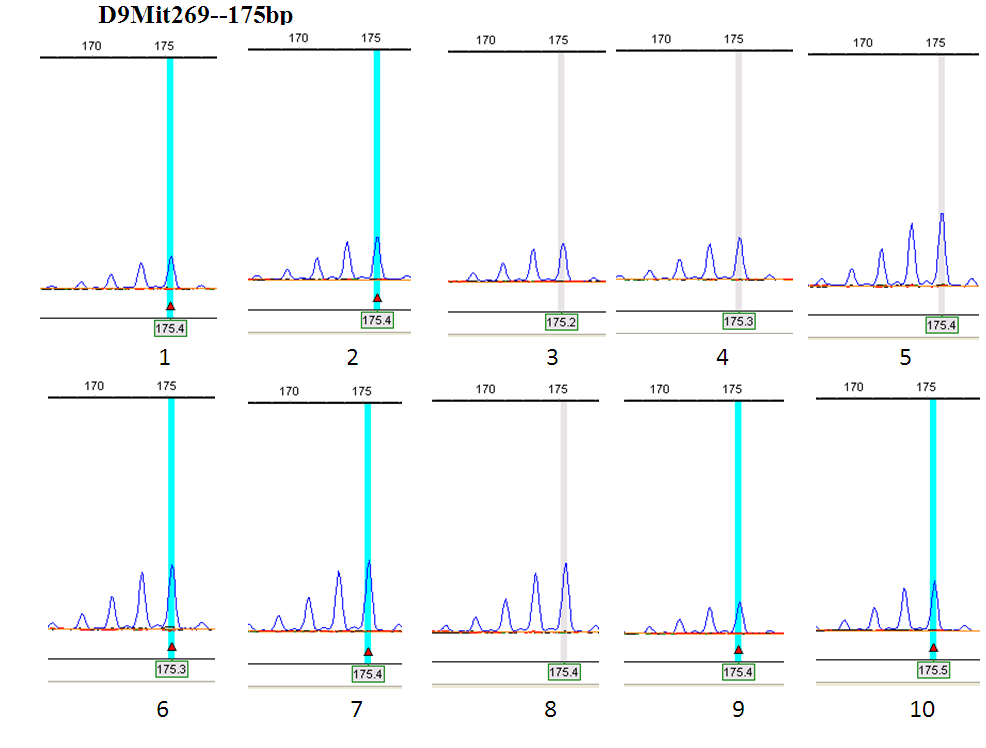

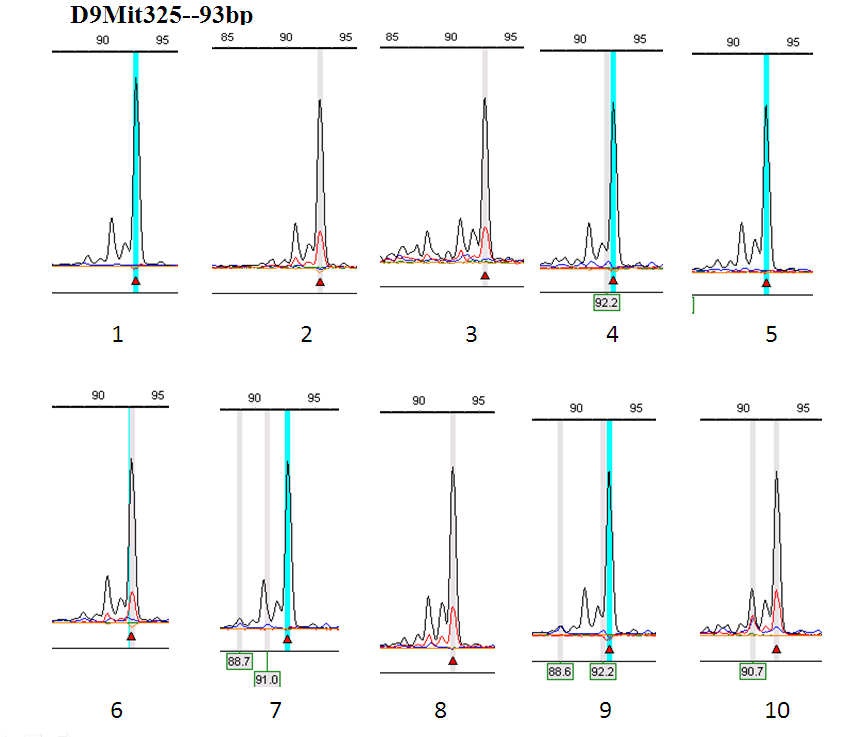

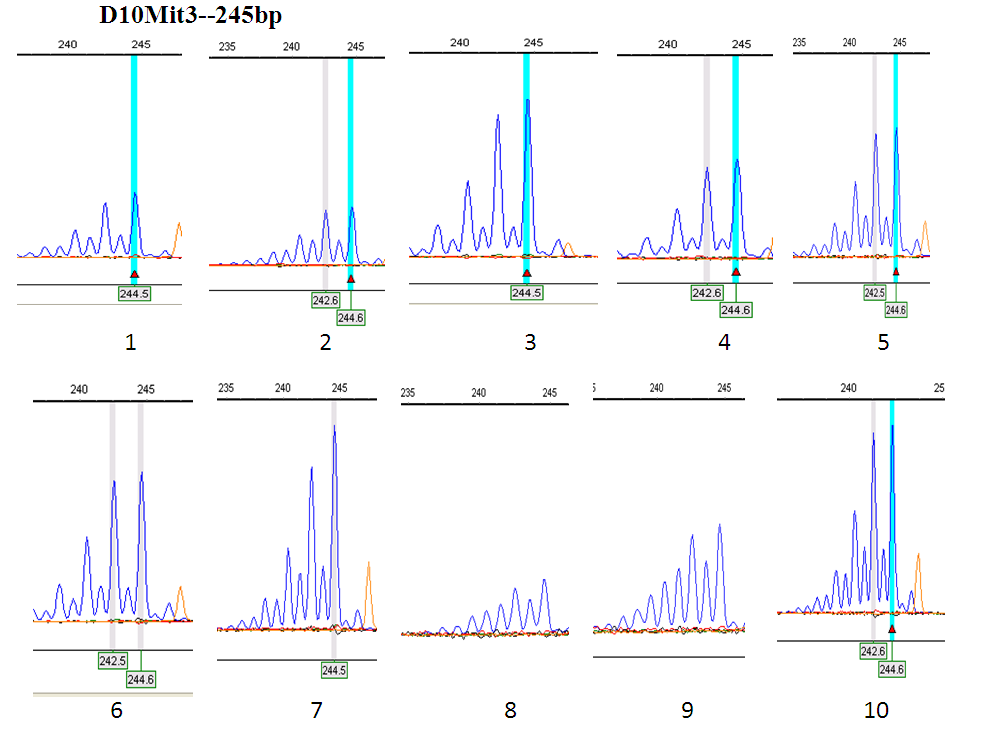

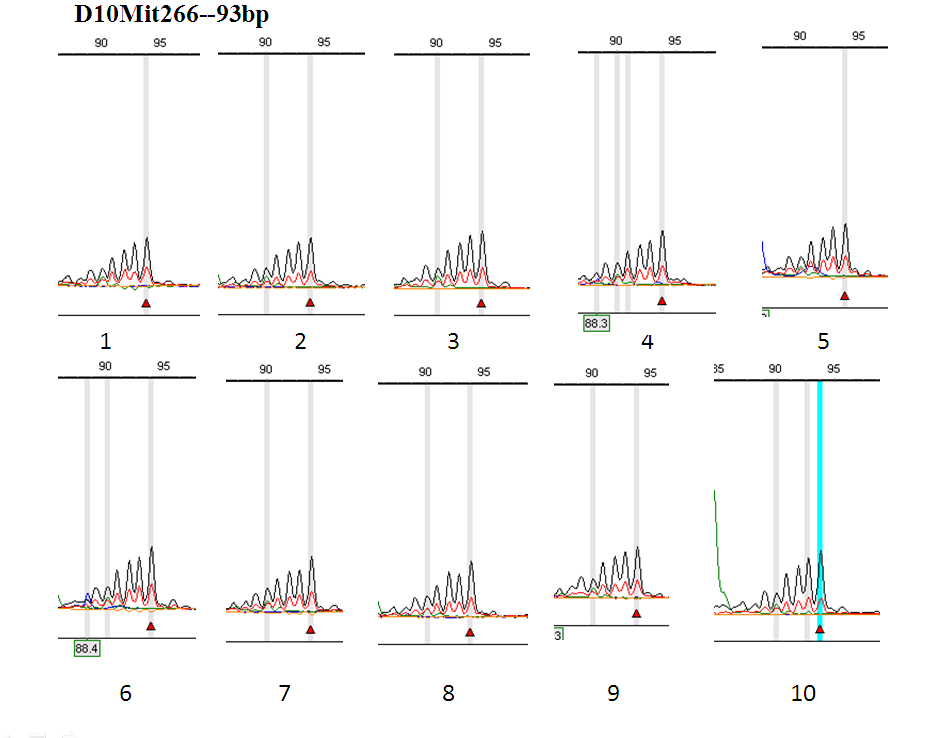

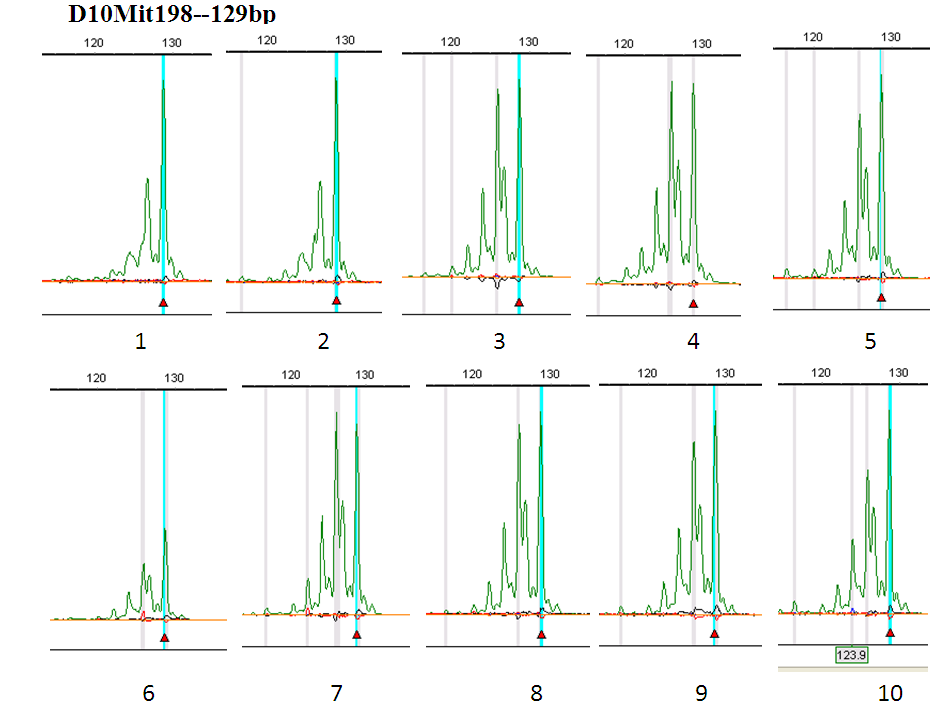

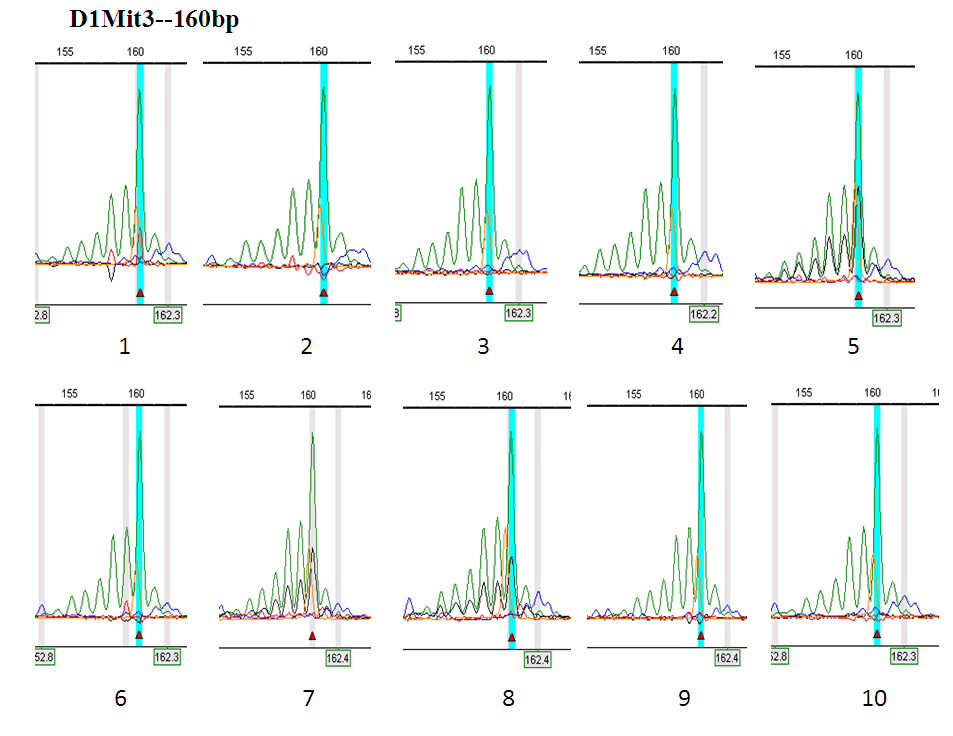

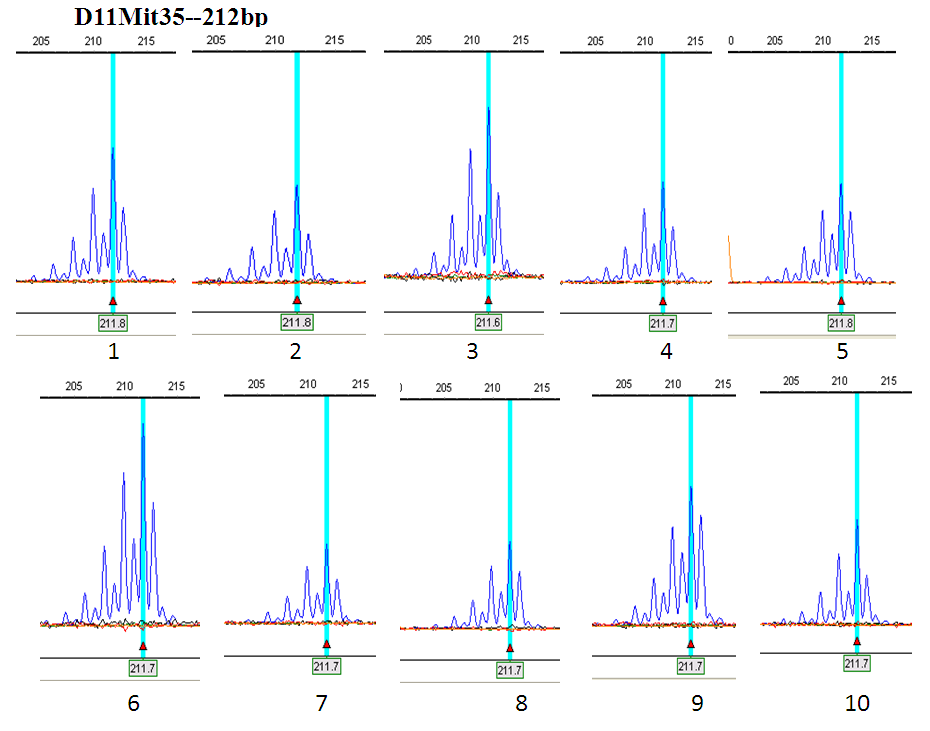

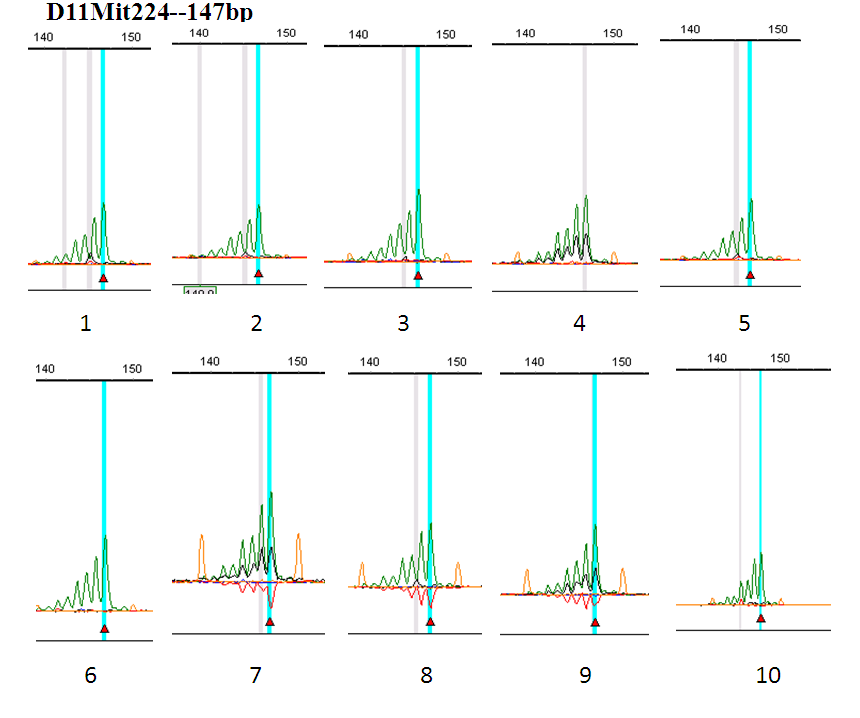

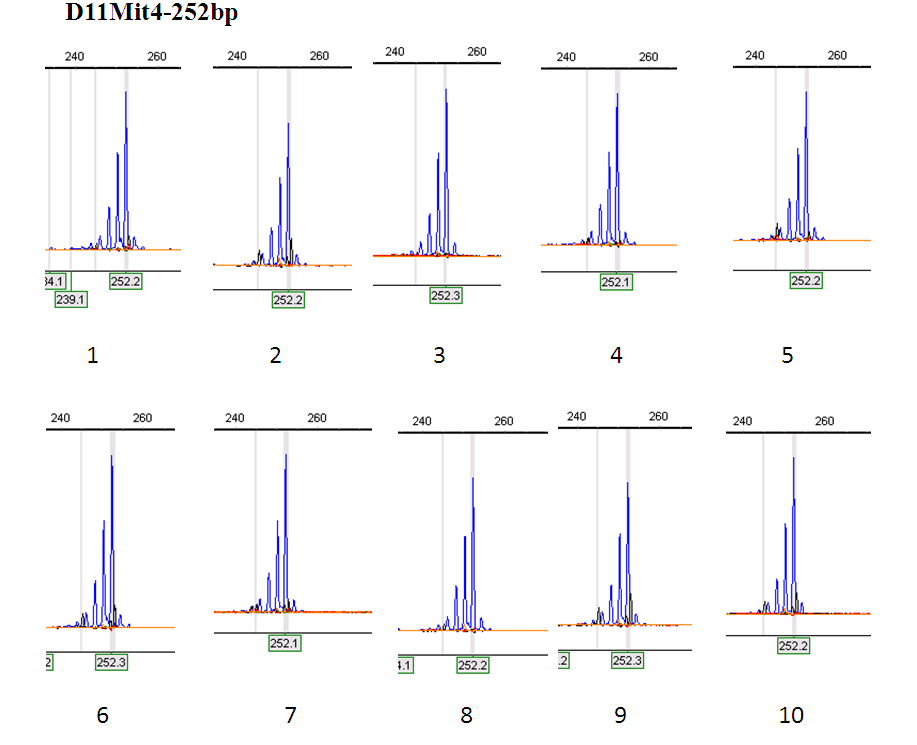

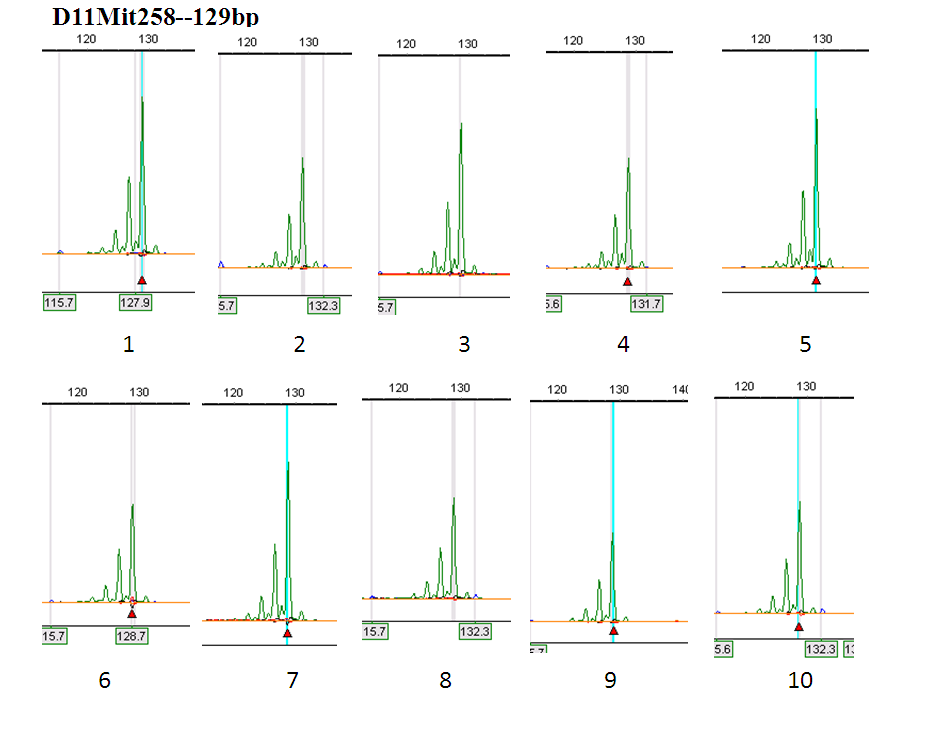

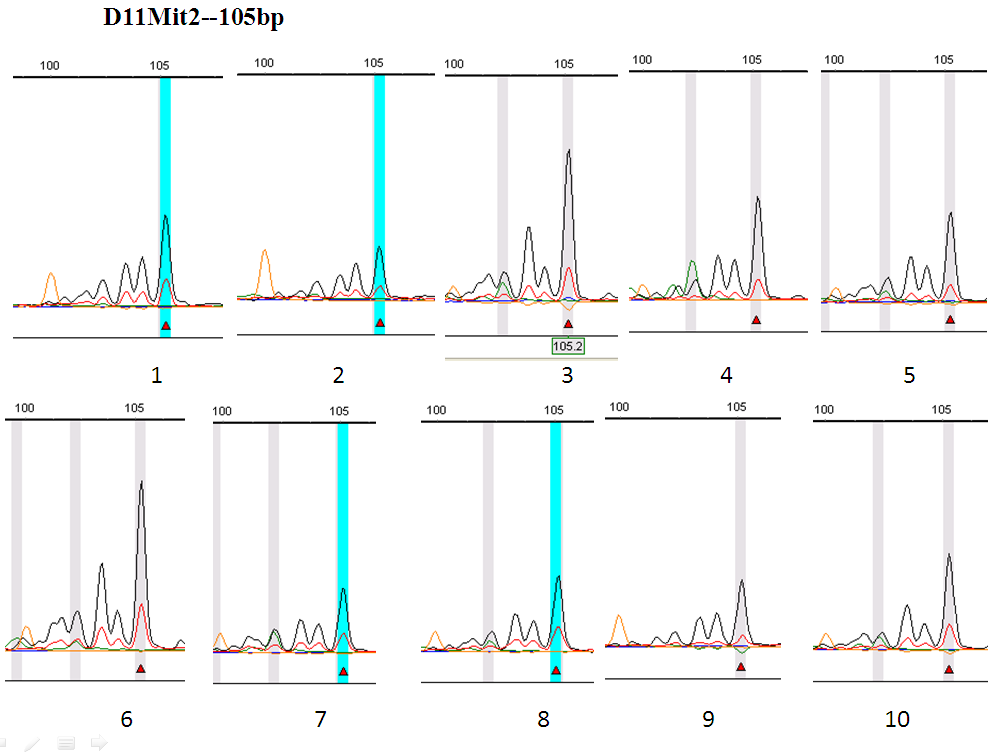

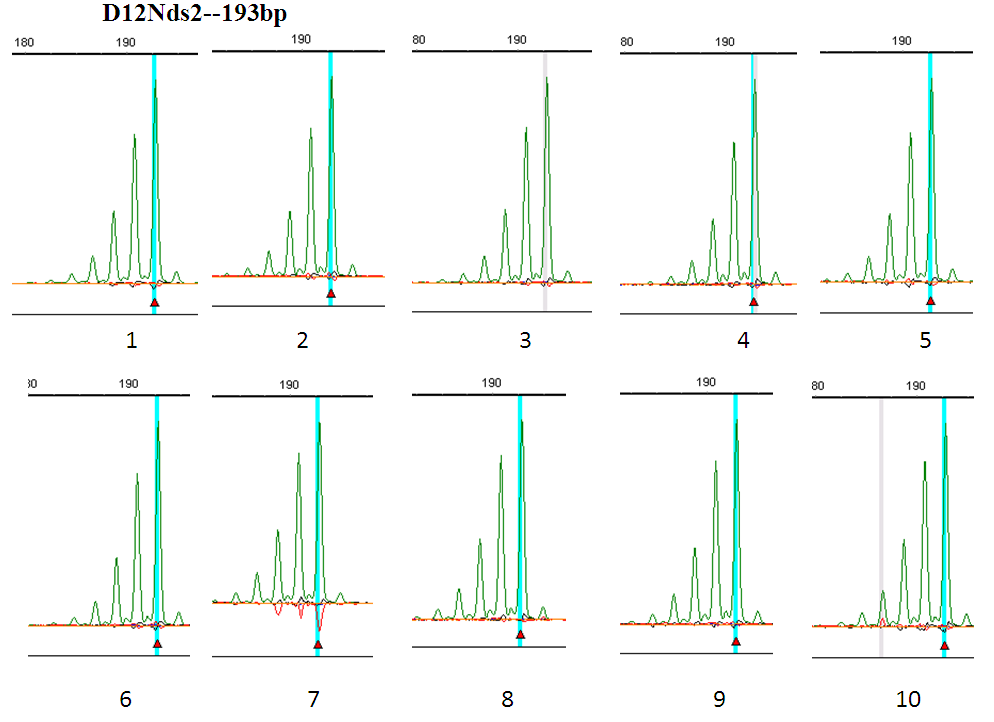

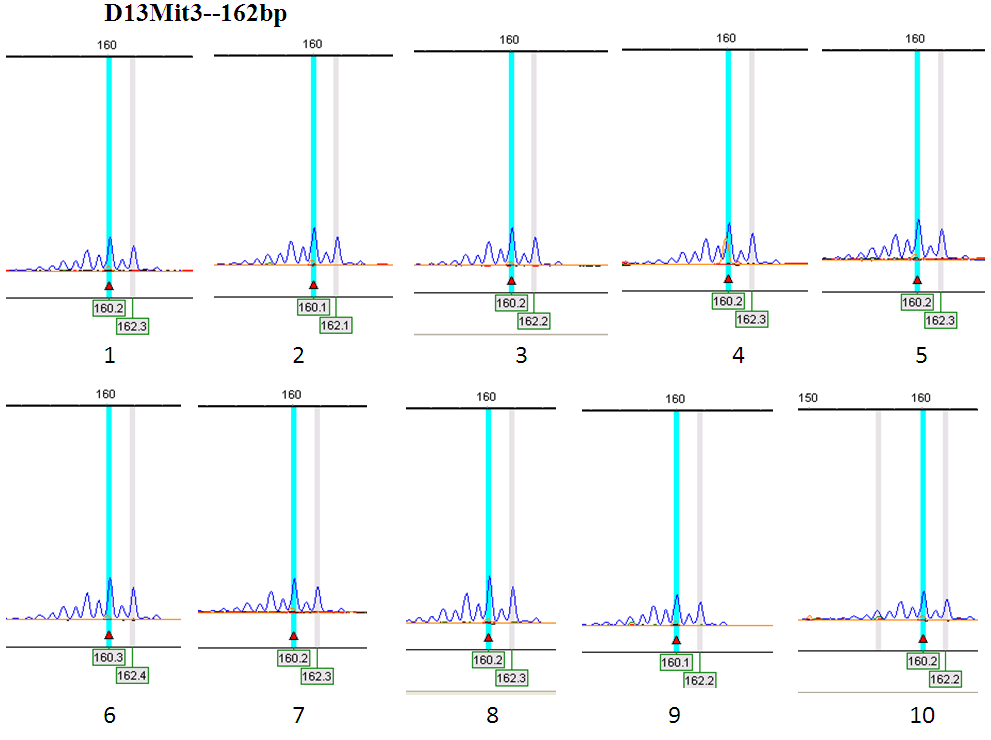

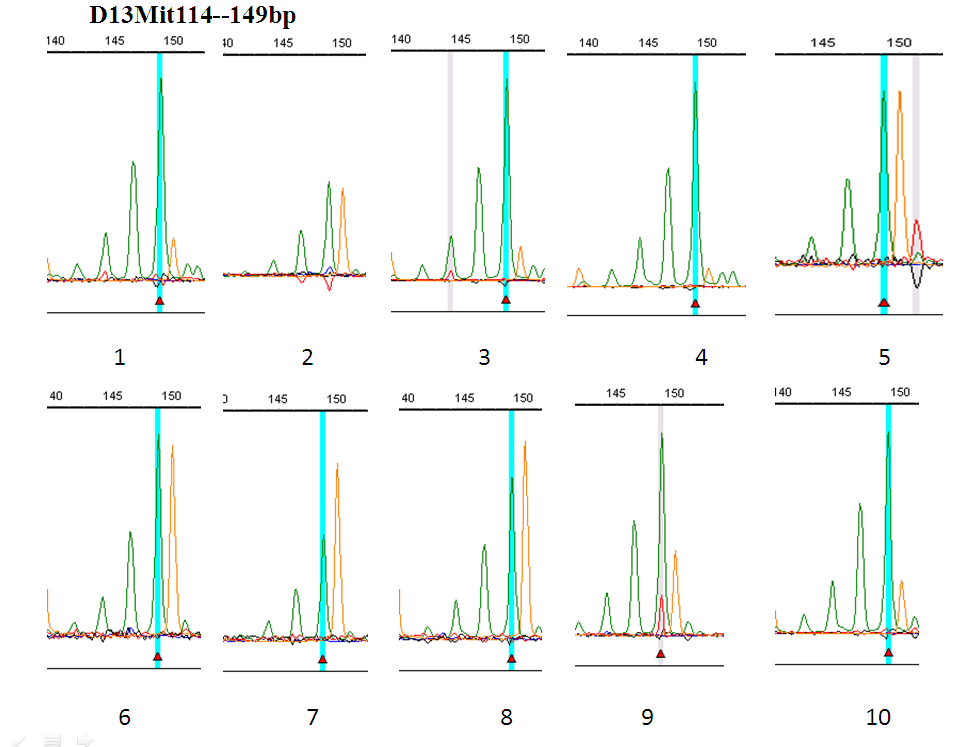

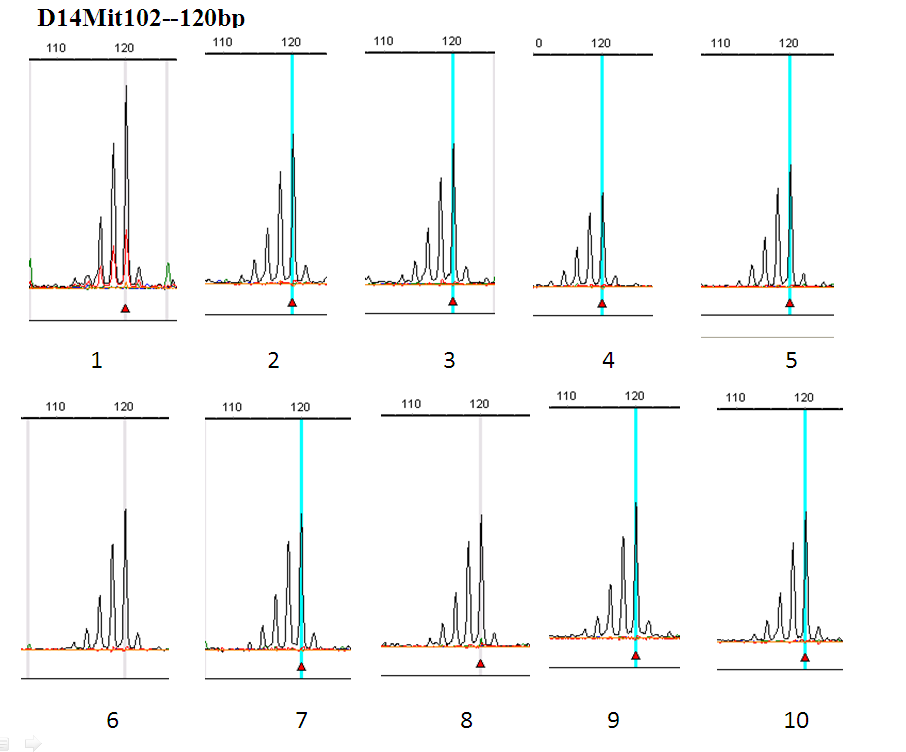

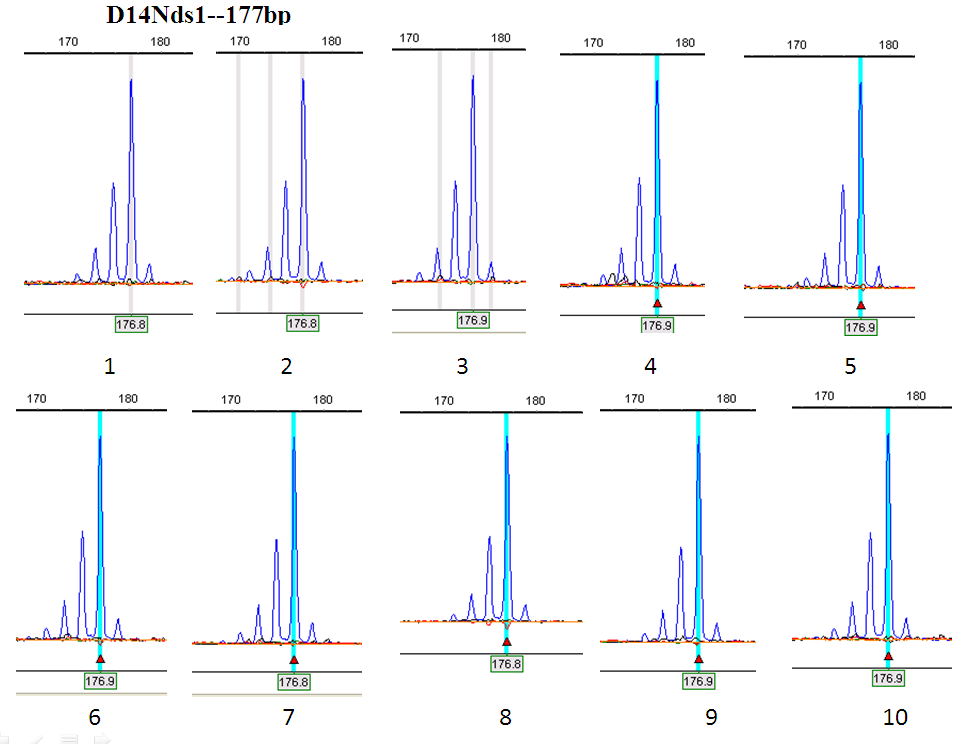

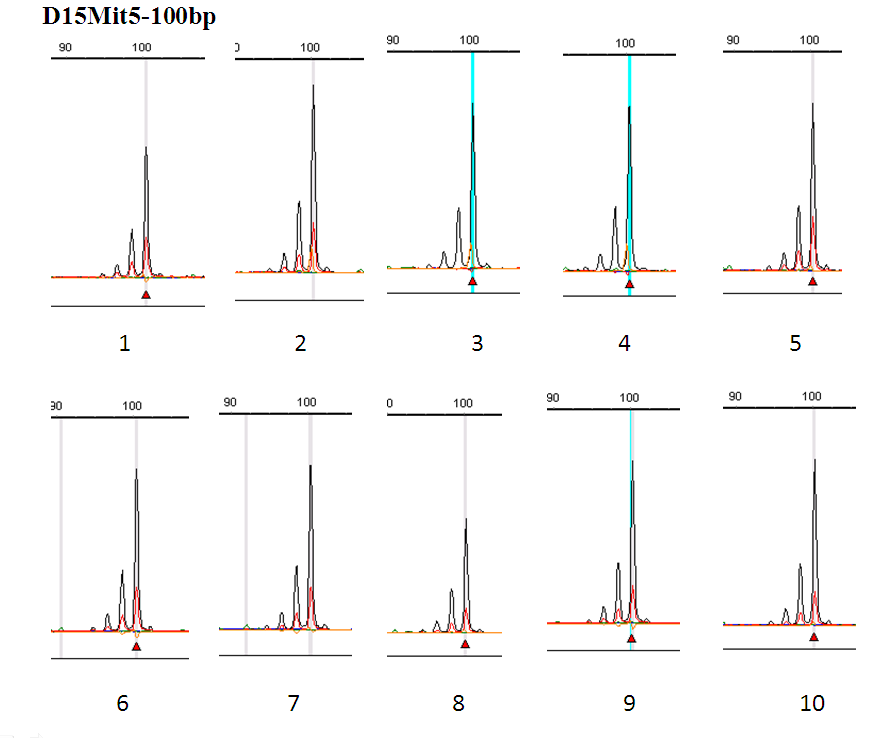

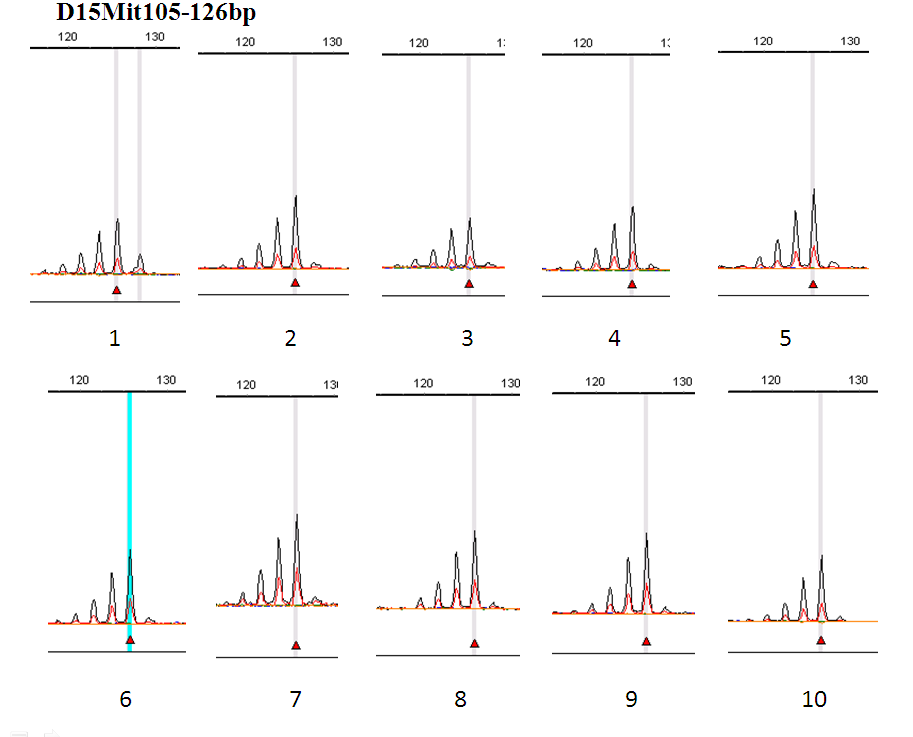

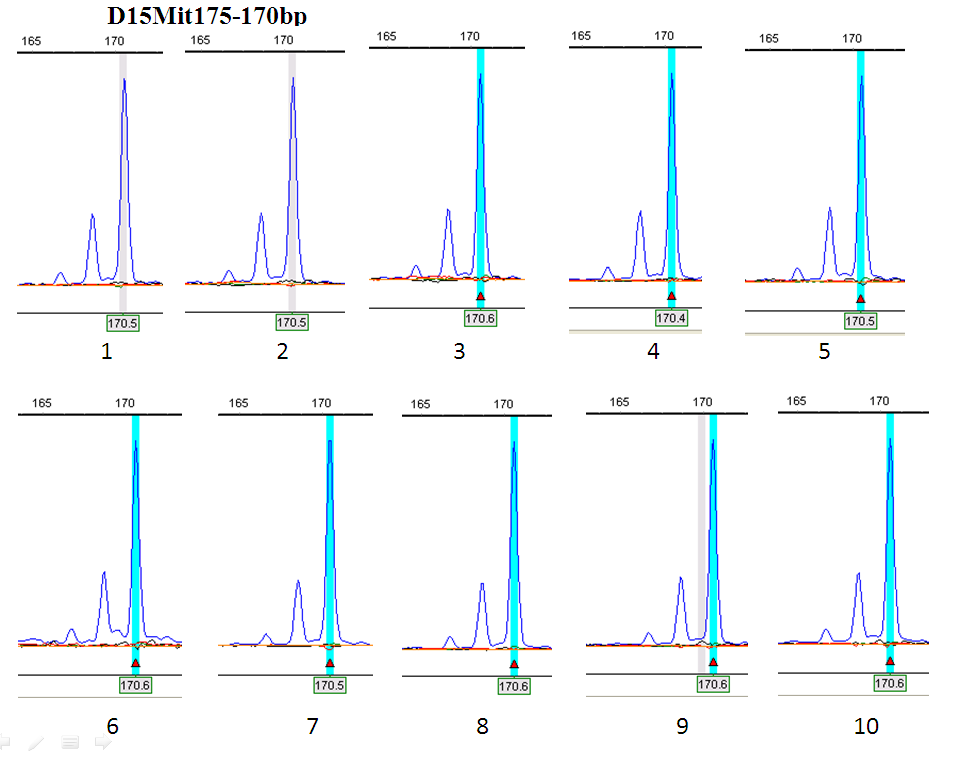

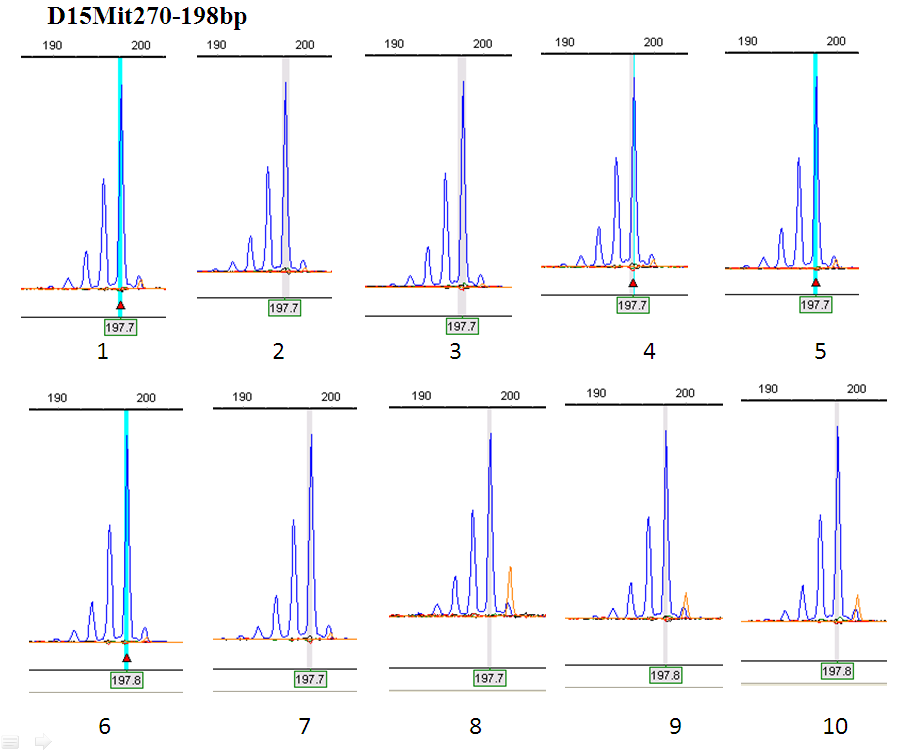

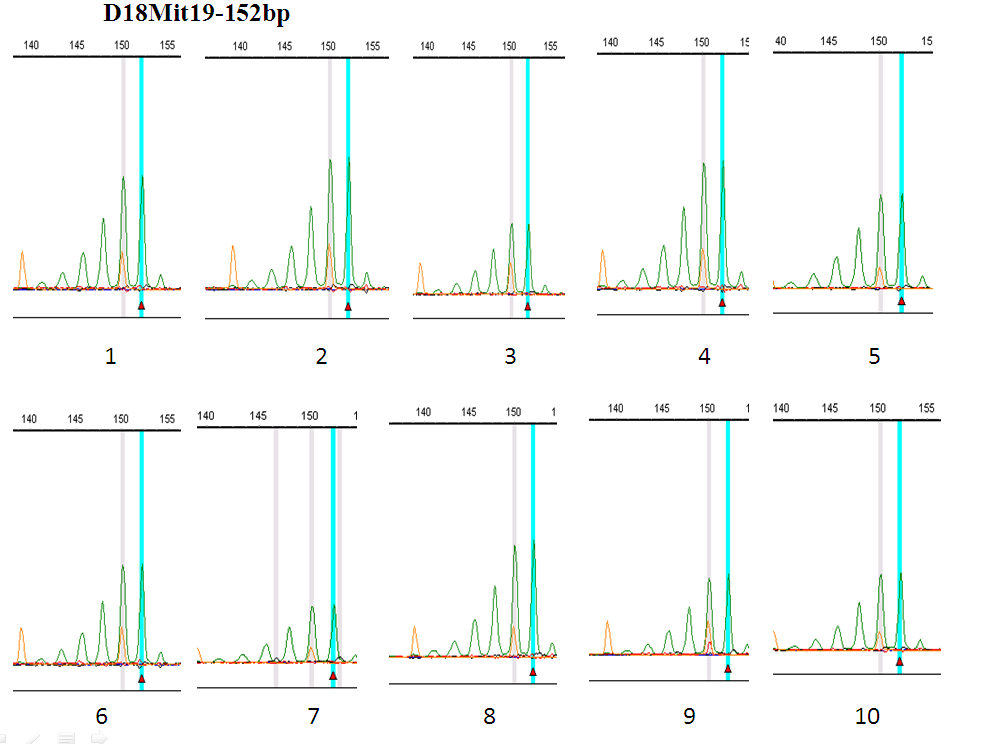

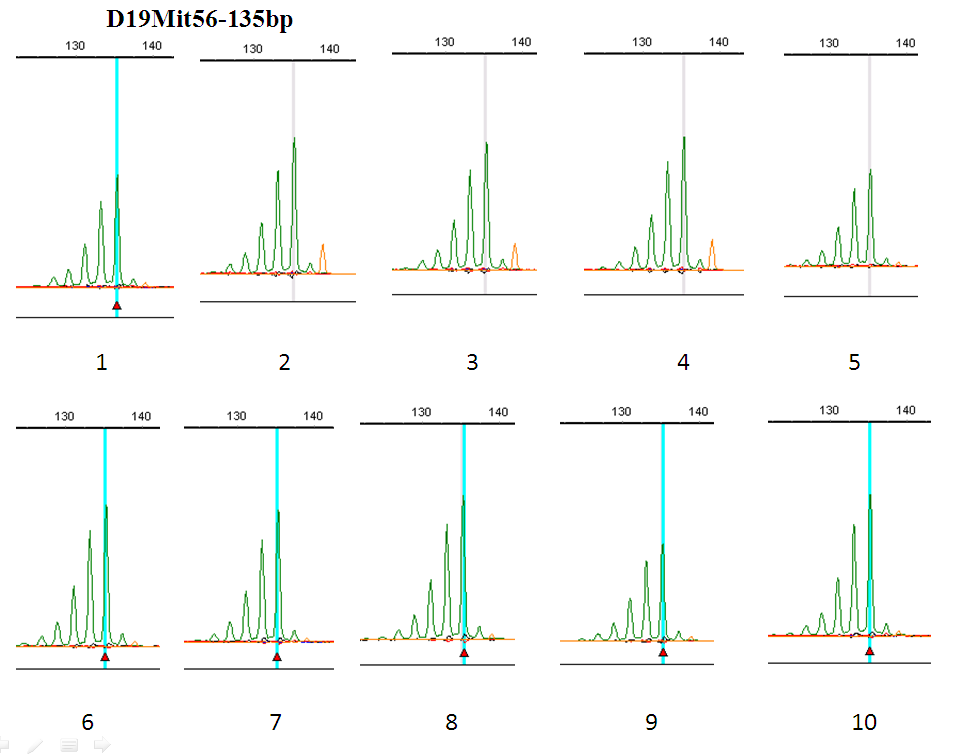

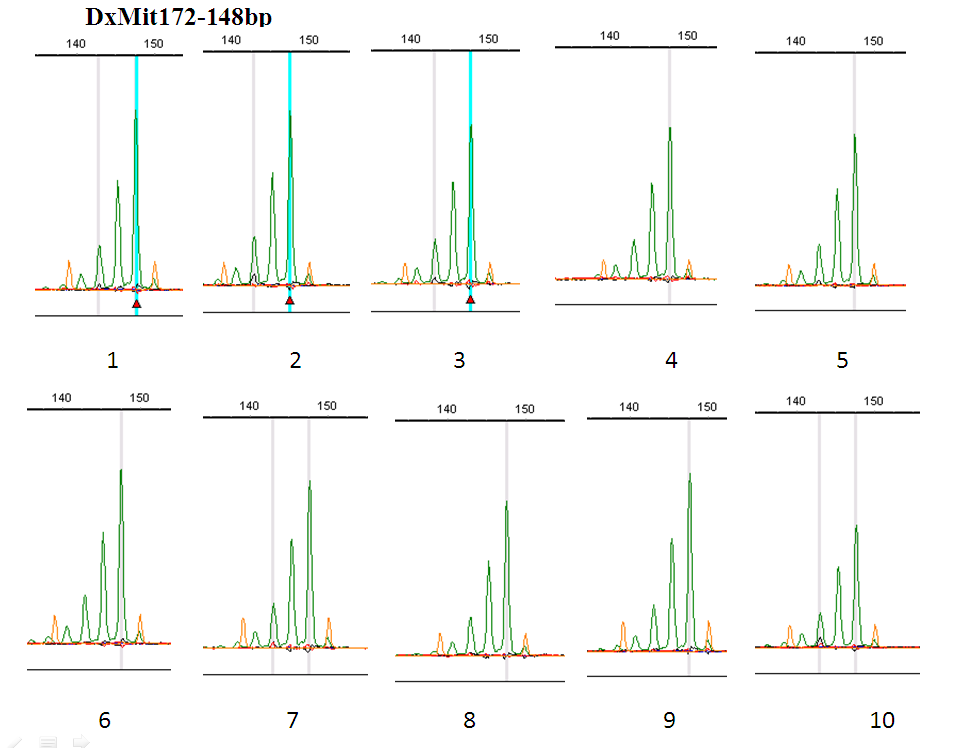
**
