# Supplementary material for: Analysis of Microsatellite Polymorphism in Inbred Knockout Mice
Source: PLoS One. 2012 Apr 11;7(4):e34555. doi: 10.1371/journal.pone.0034555 (PMC3324499; doi:10.1371/journal.pone.0034555)

Supplemental Information

Figure 2.

Mice used in this study were maintained on a normal 12 h/12h light/dark cycle with regular mouse chow and water ad libitum at an AAALAC accredited specific pathogen-free (SPF) facility. IVC cages were used for all mice within barrier facility.


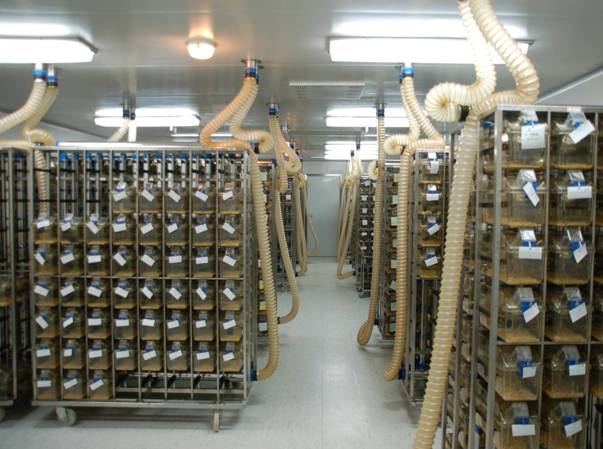

Supplement: Figure S2 — Mice used in this study were maintained on a normal 12 h/12h light/dark cycle with regular mouse chow and water ad libitum at an AAALAC accredited specific pathogen-free (SPF) facility. IVC cages were used for all mice within barrier facility. (DOC) [file pone.0034555.s002.doc]
