# Supplementary material for: Analysis of Microsatellite Polymorphism in Inbred Knockout Mice
Source: PLoS One. 2012 Apr 11;7(4):e34555. doi: 10.1371/journal.pone.0034555 (PMC3324499; doi:10.1371/journal.pone.0034555)
Supplement: Table S1 — Comparation of sequence of locus D3Mit22 between published data and ours. Green colors indicate primer sequences, red colors indicate core sequence of the locus. (DOC) [file pone.0034555.s003.doc]

**Supplemental Table 1**

Table 1. Comparation of sequence of locus D3Mit22 between published data and ours. Green colors indicate primer sequences, red colors indicate core sequence of the locus.

| Locus D3Mit22 | Size 237 bp |
| --- | --- |
| Sequence in NCBI(http://www.ncbi.nlm.nih.gov/nuccore/NT_039240.6?from=18903450&to=18903686&report=fasta  ) | 5’AATCAGCGATTTCAGCACGGAGTCTATATGTCTTTGAATGTTTCACAGTTTCTCTGAGTCAGAATGCCATTCTAAACTATAAGGCACAGTTTGGATGTGTCGACACACGGCTACGTTAGCAGCAGCAGCAGCAGCAGCAGCAGCAGCAGCAGCAGCAGCAGCAGCAGCAGCAGCAGCGGCAAAGACCACAACTCCCAACACAAGGGCAACAATTAGCCCAACCATTCTTCAATCCTT3’ |
| Sequence in our study | 3’AAGGATTGAAGAATGGTTGGGCTAATTGTTGCCCTTGTGTTGGGAGTTGTGGTCTTTGCCGCTGCTGCTGCTGCTGCTGCTGCTGCTGCTGCTGCTGCTGCTGCTGCTGCTGCTGCTGCTAACGTAGCCGTGTGTCGACACATCCAAACTGTGCCTTATAGTTTAGAATGGCATTCTGACTCAGAGAAACTGTGAAACATTCAAAGACATATAGACTCCGTGCTGAAATCGCTGATT5’ |
